# The *Staphylococcus aureus* SrrAB Regulatory System Modulates Hydrogen Peroxide Resistance Factors, Which Imparts Protection to Aconitase during Aerobic Growth

**DOI:** 10.1371/journal.pone.0170283

**Published:** 2017-01-18

**Authors:** Ameya A. Mashruwala, Jeffrey M. Boyd

**Affiliations:** Department of Biochemistry and Microbiology, Rutgers University, New Brunswick, New Jersey, United States of America; National Institute of Child Health and Human Development, UNITED STATES

## Abstract

The SrrAB two-component regulatory system (TCRS) positively influences the transcription of genes involved in aerobic respiration in response to changes in respiratory flux. Hydrogen peroxide (H_2_O_2_) can arise as a byproduct of spontaneous interactions between dioxygen and components of respiratory pathways. H_2_O_2_ damages cellular factors including protein associated iron-sulfur cluster prosthetic groups. We found that a *Staphylococcus aureus* strain lacking the SrrAB two-component regulatory system (TCRS) is sensitive to H_2_O_2_ intoxication. We tested the hypothesis that SrrAB manages the mutually inclusive expression of genes required for aerobic respiration and H_2_O_2_ resistance. Consistent with our hypothesis, a Δ*srrAB* strain had decreased transcription of genes encoding for H_2_O_2_ resistance factors (*kat*, *ahpC*, *dps*). SrrAB was not required for the inducing the transcription of these genes in cells challenged with H_2_O_2_. Purified SrrA bound to the promoter region for *dps* suggesting that SrrA directly influences *dps* transcription. The H_2_O_2_ sensitivity of the Δ*srrAB* strain was alleviated by iron chelation or deletion of the gene encoding for the peroxide regulon repressor (PerR). The positive influence of SrrAB upon H_2_O_2_ metabolism bestowed protection upon the solvent accessible iron-sulfur (FeS) cluster of aconitase from H_2_O_2_ poisoning. SrrAB also positively influenced transcription of *scdA* (*ytfE*), which encodes for a FeS cluster repair protein. Finally, we found that SrrAB positively influences H_2_O_2_ resistance only during periods of high dioxygen-dependent respiratory activity. SrrAB did not influence H_2_O_2_ resistance when cellular respiration was diminished as a result of decreased dioxygen availability, and negatively influenced it in the absence of respiration (fermentative growth). We propose a model whereby SrrAB-dependent regulatory patterns facilitate the adaptation of cells to changes in dioxygen concentrations, and thereby aids in the prevention of H_2_O_2_ intoxication during respiratory growth upon dixoygen.

## Introduction

*Staphylococcus aureus* is a human pathogen that has the ability infect nearly every tissue of the body [1]. The ability of *S*. *aureus* to sense various environmental stimuli, and rapidly calibrate its cellular physiology in response, is a cornerstone of its success as a pathogen. *S*. *aureus* is a facultative anaerobe and can respire dioxygen. Hydrogen peroxide (H_2_O_2_) is a deleterious by-product of aerobic respiration, and can arise as a result of interactions between dioxygen and components of respiratory pathways [2–5]. H_2_O_2_ can cause damage to cellular membranes and biological polymers, as well as oxidize protein bound iron-sulfur (FeS) cluster prosthetic groups [6, 7]. Oxidation of FeS clusters by H_2_O_2_ results in cluster disintegration and enzyme inactivation [7–9]. Ultimately, H_2_O_2_ exposure can result in metabolic standstill and eventually cell death [10, 11]. The importance of reactive oxygen species (ROS) in preventing *S*. *aureus* infections is evidenced by the fact that individuals carrying metabolic or genetic defects affecting ROS formation by polymorphonuclear neutrophils, the first line of defense in human innate immunity, often have chronic and reoccurring *S*. *aureus* infections [12].

The *S*. *aureus* genome encodes for a variety of mechanisms to detoxify ROS and to repair the damage caused by ROS to cellular molecules. Staphylococcal cells maintain high titers of the H_2_O_2_ scavenging enzymes catalase (Kat), alkylhydroperoxidase (Ahp) and the superoxide scavengers SodA and SodM [13–15]. In contrast to *Escherichia coli*, Kat is proposed to be the primary intracellular H_2_O_2_ scavenger in *S*. *aureus* [16]. SodA is proposed to be the dominant aerobic superoxide dismutase [15, 17, 18]. The findings that strains lacking ROS-scavenging enzymes display attenuated virulence in models of infection underscores the value of these ROS scavenging mechanisms [16, 19]. H_2_O_2_ can oxidize ferrous iron resulting in the production of hydroxyl radicals (Fenton reaction), which readily damage DNA [20]. The effects of Fenton chemistry are suppressed by producing a bi-functional protein (Dps) that binds and sequesters iron, as well as binds and protects DNA from oxidative damage [21–23]. *S*. *aureus* also encodes for a di-iron RIC protein ScdA (YtfE in *Escherichia coli*) that has a role in repairing FeS proteins damaged by H_2_O_2_ [24, 25]_._

The genome of *S*. *aureus* encodes for sixteen two-component regulatory systems (TCRS). TCRS permit bacteria to integrate multiple signals into cellular signaling circuits allowing for a rapid and robust response to stimuli [26]. TCRS typically consist of two proteins: a histidine kinase (HK) and a response regulator (RR). The HK can be either cytosolic or membrane-associated and the RR is typically a cytosolic DNA binding protein. A phosphorylation cascade between the HK and RR results in conformational changes in the RR protein, altered affinity towards target DNA sequences, modified gene expression, and a tailored physiological response [27]. TCRS have been implicated in the oxidative stress response in multiple bacterial species [28–30].

The staphylococcal respiratory regulator (SrrAB) is a TCRS that is thought to be the dominant transcriptional regulator mediating the aerobic to anaerobic switch in *S*. *aureus* [31–34]. SrrAB is a pleiotropic regulator of aerobic and anaerobic respiration and energy metabolism and is capable of activating as well as repressing gene transcription [31, 34–37]. SrrAB also modulates the response to nitric oxide stress [36, 38]. Alterations in cellular respiratory flux serve as one stimulus for SrrAB [36]. *S*. *aureus* strains lacking SrrAB display attenuated survival in models of infection [34, 38, 39].

In this study we focused upon defining a role for the SrrAB TCRS in mediating resistance to H_2_O_2_. Previous studies found that SrrAB positively influences expression of multiple genes required for aerobic respiration and changes in cellular respiratory flux act as a stimulus for SrrAB [34, 36]. We tested a model wherein *S*. *aureus* use SrrAB to co-regulate genes required for aerobic respiration and H_2_O_2_ resistance. Such a regulatory tuning mechanism would allow respiring cells to achieve physiological homeostasis by protecting and repairing cellular macromolecules from H_2_O_2_ damage. In support of this model, SrrAB positively influenced the transcription of genes involved in H_2_O_2_ resistance and aerobic respiration (*ahpC*, *kat*, *dps*, *scdA*, *cydB*) during respiratory growth. However, SrrAB was not required for inducing the transcription of H_2_O_2_ resistance genes upon challenge with H_2_O_2_. Purified SrrA bound to the promoter region for *dps* suggesting that SrrAB directly modulates transcription of at least one H_2_O_2_ resistance factor. Intriguingly, SrrAB negatively modulated H_2_O_2_ resistance during conditions of low respiration or in its absence. During respiratory growth the positive influence of SrrAB upon H_2_O_2_ resistance bestowed protection upon the solvent accessible FeS cluster of aconitase from ROS poisoning.

## Material and Methods

### Materials

Restriction enzymes, quick DNA ligase kit, deoxynucleoside triphosphates, and Phusion DNA polymerase were purchased from New England Biolabs. The plasmid mini-prep kit, gel extraction kit, and RNAprotect were purchased from Qiagen. DNase I was purchased from Ambion. Lysostaphin was purchased from Ambi products. Oligonucleotides were purchased from Integrated DNA Technologies and sequences are listed in S1 Table. Trizol and High-Capacity cDNA Reverse Transcription Kits were purchased from Life Technologies. Tryptic Soy broth (TSB) was purchased from MP biomedical. Unless specified, all chemicals were purchased from Sigma-Aldrich and were of the highest purity available.

### Bacterial growth conditions

Unless otherwise stated, the *S*. *aureus* strains used in this study (Table 1) were constructed in the *S*. *aureus* community-associated USA300_LAC strain that was cured of the native plasmid pUSA03, which confers erythromycin resistance (USA300_LAC or WT) [40]. *S*. *aureus* were cultured overnight aerobically in 10 mL culture tubes with a culture volume of 1 mL. For subcultures in the presence of dioxygen, overnight cultures were used to inoculate either 10 or 30 mL culture tubes covered with a loosely fitting stainless-steel cap (Kap-Uts) that allowed for free diffusion of gases. Unless otherwise indicated, the 10 mL tubes contained 1 mL of media and the 30 mL tubes contained 5 mL of media. Liquid shake-tube cultures were grown at 37°C with shaking at 250 rpm unless otherwise indicated. Aerobic phenotypic analyses in 96-well plates were assessed in 200 μL cultures using a BioTek 808E visible absorption spectrophotometer with medium shake speed and the culture optical density was monitored at 600 nm. Difco agar was added (15 g L^-1^) for solid medium. The staphylococcal defined media contained per 100 mL: 1 g (NH_4_)_2_SO_4_, 4.5 g KH_2_PO_4_, 10.5 g K_2_HPO_4_, 110 mM NaCl, 30 mM KCl, 50 μg nicotinic acid, 50 μg thiamine, 0.3 μg biotin and 0.025 mg of each of the 20 canonical amino acids. Glucose (14 mM) was added as carbon sources. When required antibiotics were added at the following concentrations: 150 μg mL^-1^ ampicillin; 30 μg mL^-1^ chloramphenicol (Cm); 10 μg mL^-1^ erythromycin (Erm); 3 μg mL^-1^ tetracycline (Tet); 125 μg mL^-1^ kanamycin (Kan); 150 ng mL^-1^ anhydrotetracycline (Atet). To maintain plasmids media was supplemented with either 15 μg mL^-1^ or 5 μg mL^-1^ of chloramphenicol or erythromycin, respectively.

**Table 1:** Strains and plasmids used in this study.

*S. aureus Strains*	Genotype/Description	Genetic Background	Source/Reference
JMB1100	USA300_LAC (erm sensitive)	LAC	[40]
RN4220	Restriction minus	NCTC8325	[86]
JMB 1467	Δ*srrAB* (SAUSA300_1441–42)	LAC	[87]
JMB 2047	Δ*srrAB*::*tet*	LAC	This work
JMB 1209	Δ*dps* (SAUSA300_2092)	LAC	This work
JMB6326	*sodA*::*Tn* (*ermB*) (SAUSA300_1513)	LAC	NARSA [88]
JMB 2968	*sodM*::*Tn* (*ermB*) (SAUSA300_0135)	LAC	NARSA [88]
JMB2080	Δ*ahpC*::*erm* (SAUSA300_0379)	LAC	V. Torres
JMB 2078	Δ*kat*::*erm* (SAUSA300_1232)	LAC	V. Torres
JMB2151	Δ*perR*::*kan* (SAUSA300_1842)	LAC	V. Torres
JMB2615	Δ*srrAB* Δ*perR*::*kan*	LAC	This work
JMB7103	Δ*srrAB* Δ*ahpC*::*erm*	LAC	This work
JMB 5562	Δ*srrAB* Δ*kat*::*erm*	LAC	This work
JMB6069	*cidA*::*Tn* (*ermB*) (SAUSA300_2479)	LAC	[88]
JMB6070	Δ*srrAB cidA*::*Tn* (ermB)	LAC	This work
JMB 6023	*cidB*::*Tn* (*ermB*)	LAC	NARSA [88]
JMB 6024	Δ*srrAB cidB*::*Tn* (*ermB*) (SAUSA300_2479)	LAC	This work
SH1000	parent	SH1000	[77]
JMB4556	Δ*srrAB*::*tet*	SH1000	This work
Newman	parent	Newman	[89]
JMB 4751	Δ*srrAB*::*tet*	Newman	This work
JMB 2030	Δ*srrAB*::*tet*	RN4220	This work
JMB1432	*fur*::*tet*	LAC	[90]
JMB 1163	*acn*::*tet*	LAC	[91]
JMB 7105	Δ*ahpC*::*erm acn*::*tet*	LAC	This work
JMB 3537	*acn*::*Tn erm*	LAC	NARSA [88]
JMB 3538	Δ*nfu acn*::*erm*	LAC	This work
JMB 7107	Δ*kat*::*erm acn*::*tet*	LAC	This work
JMB 4367	Δ*srrAB acn*::*erm*	LAC	This work
JMB1254	Δ*scdA* (SAUSA300_0253)	LAC	This work
**Other Strains**			
*Escherichia coli* PX5			Protein Express
*Escherichia coli* BL21-AI			Life Technologies
**Plasmids used in this study**			
**Plasmid name**	**Insert Locus/function**		**Source/Reference**
pJB38	construction of chromosomal gene deletions		[92]
pJB38_Δ*scdA*	Construction of Δ*scdA*		This work
pJB38_Δ*dps*	Construction of Δ*dps*		This work
pJB38_Δ*srrAB*::*tet*	Construction of *srrAB*::*tet* allele		This work
pCM28	Cloning vector for genetic complementation		A. Horswill
pCM11	Cloning vector for trascriptional reporters		[45]
pCM11_P*sufC*	Reporter construct transcriptional activity		[46]
pCM11_P*dps*	Reporter construct transcriptional activity		This work
pCM28_*srrAB*	Complementing vector		This work
pEPSA5	Xylose inducible over-expression		[93]
pEPSA5_*acnA* (*pacnA*)	Aconitase over-production		[47, 94]
pET20b	Cloning vector for protein production		EMD Millipore
pET20b(+)_*srrA*	Protein production		This work

### Growth analyses for hydrogen peroxide resistance

Strains were cultured overnight in TSB at the indicated culture vessel headspace to the medium volume ratio (HVR). Optical density (OD) on all strains was standardized to OD 2.5 (A_600_) in a final volume of 1 mL of 1X PBS. Two μL of the resuspended culture was used to inoculate 198 μL media in a 96-well microtiter plate. A multichannel pipettor was used to amend the media with hydrogen peroxide or vehicle control and the cultures were rapidly mixed. Bacterial strains were then cultured with constant shaking in a heated microplate reader.

#### Challenge during early exponential growth

Strains cultured overnight in TSB were diluted into fresh TSB medium to a final OD of 0.1 (A_600_) at a HVR of 10 and allowed to grow with shaking for two doublings (OD of 0.4 (A_600_)). Subsequently, the strains were challenged with H_2_O_2_ or vehicle control as outlined above.

#### Challenge during fermentative (anaerobic) growth

Strains were cultured overnight in TSB at a HVR of 0, as described earlier [41–43]. At point of harvest the cultures tubes were placed inside a COY anaerobic chamber (<1 ppm of O_2_). Optical density on all strains was standardized in 1X PBS. The strains were then removed from the COY chamber and immediately sub-cultured into fresh TSB medium and challenged with H_2_O_2_ or vehicle control as outlined above.

### H_2_O_2_ killing assays

Bacterial strains were cultured with shaking to OD 10 (A_600_) in TSB. Cells were pelleted by centrifugation and resuspended in an equal volume of PBS. The optical density for all the strains was adjusted to an OD of 0.7 (A_600_) in a total volume of 1 mL of 1X PBS. Cells were subsequently challenged with a bolus of H_2_O_2_ and incubated for two hours at room temperature. Fifty μL of the reaction mixture was diluted 1:20 into PBS buffer containing catalase (1300 units mL^-1^). Colony forming units (CFU) were determined by serially diluting cells and spot plating upon TSB agar plates.

### Recombinant DNA and genetic techniques

All clones were passaged through RN4220 and subsequently transduced into the appropriate strains using bacteriophage 80α [44]. All *S*. *aureus* mutant strains and plasmids were verified using PCR or by sequencing PCR products or plasmids. DNA sequencing was performed at Genewiz, (South Plainfield, NJ).

### Creation of plasmids and mutant strains

Chromosomal DNA from JMB 1100 was used as the template for PCR reactions for the construction of plasmids. To create the *scdA* chromosomal deletion, approximately 500 base pairs upstream and downstream of the *scdA* gene (SAUSA300_0253) was amplified using PCR the following primer pairs; 0253up5EcoRI and up3NheI; down5MluI and down3KpnI. Amplicons were gel purified and joined by PCR using the 0253up5EcoRI and down3KpnI primer pair. The amplicon was digested with EcoRI and KpnI, and ligated into similarly digested pJB38 [10]. The ligation was transformed into *E*. *coli* DH5α and colonies were screened by PCR for the correct plasmid. Plasmid pJB38_Δ*scdA* was isolated and transformed into RN4220 by selection upon TSA-Cm at 30°C. Plasmid pJB38_Δ*scdA* was transduced into JMB1100, and single colonies were inoculated into 5 mL of TSB-Cm. Cultures were grown at 42°C overnight before plating upon TSA-Cm to select for single recombinants. Single colonies were inoculated into 5 mL of TSB medium, grown overnight, and cultures were diluted 1:25,000 before plating 100 μL on TSA-anhydrotetracycline to select for the loss of plasmid. Cm sensitive colonies were screened for the double recombination event using PCR with primers 0253up5EcoRI and down3KpnI.

To create the *dps* chromosomal deletion, approximately 500 base pairs upstream and downstream of the *dps* gene (SAUSA300_2092) was amplified using PCR the following primer pairs; 2092up5EcoRI and 2092up3NheI; 2092down5MluI and 2092down3SalI. Amplicons were gel purified and joined by PCR using the 2092up5EcoRI and 2092down3SalI primer pair. The amplicon was digested with EcoRI and SalI, and ligated into similarly digested pJB38. Thereafter the Δ*dps* strain was created as outlined above.

The pJB38_Δ*srrAB*::*tet* plasmid was created by using PCR to amplify the *tetM* allele from strain JMB1432 using primers G+tetMluI and G+tetNheI. The PCR product was digested with MluI and NheI and ligated into similarly digested pJB38_Δ*srrAB* (pJB38_Δ*srrAB*::*tetM*). The Δ*srrAB*::*tetM* strain was created as outlined above.

Transcriptional reporter plasmids for *sufC* and *dps* were constructed using the pCM11 vector backbone [45, 46]. Primers were designed to amplify the upstream regions (200–800 base pairs) upstream of the annotated translational start site for the genes. PCR amplicons were digested with HindIII and KpnI and subsequently ligated into similarly digested plasmid.

### Quantitative real-time PCR assays

Overnight cultures were diluted to an OD of 0.1 (A_600_) in a final volume of 5 mL of fresh TSB (HVR 6) and cDNA libraries were constructed as previously described [47].

#### ROS challenge

Culture optical density was standardized to an OD of 0.1 (A_600_) in a final volume of 5 mL of fresh TSB (HVR 6) and strains were subsequently grown with shaking to the desired optical densities and treated with either 10 mM H_2_O_2_ or the vehicle control and grown for an additional 25 minutes. Aliquots of cells were then harvested by centrifugation and treated with RNAprotect (Qiagen) reagent prior to RNA extraction and cDNA library preparation.

### Anaerobic work

Unless otherwise mentioned anaerobic work was performed using a Coy anaerobic glove-box (Grass Lake, MI). Solutions, plastic-ware, and liquid and solid growth medium was allowed to equilibrate for >6 h inside the glove-box before use.

### Cell-free extract enzyme assays

#### Aconitase (AcnA) assays

Overnight cultures were diluted to a final OD of 0.1 (A_600_) in fresh TSB growth at HVR of 2.5, 5, 10 or 20 (ratios were altered as per experimental requirement) in the presence or absence of 1% xylose. Strains were cultured to an OD of 8 (A_600_) and cell pellets were harvested by centrifugation. Cell-free lysates/extracts were prepared inside a COY anaerobic chamber using anaerobic lysis buffer (25 mM Tris, 150 mM NaCl, pH 7.4), as described previously [47]. The assay was initiated by addition of 25 μL of lysate to 975 μL of lysis buffer containing 20 mM DL-isocitrate. Aconitase activity was determined by monitoring the conversion of isocitrate to cis-aconitate spectrophotometrically using a Beckman Coulter DU530 UV-Vis absorption spectrophotometer (cis-aconitate ε240 nm = 3.6 mM^-1^cm^-1^ [48]).

For AcnA assays under anaerobic conditions, strains were cultured in 2 mL microcentrifuge tubes containing TSB and 1% xylose at a headspace to volume ratio of zero, as described earlier [47].

The effect of dioxygen on AcnA activity was assessed as described earlier [42]. Briefly, strains were cultured anaerobically as described above. Protein synthesis was inhibited by the addition of anaerobic 100 μg mL^-1^ rifampicin inside a COY chamber. Dioxygen was introduced to one set of cultures by decanting into tubes at a HVR of 15 and vigorous shaking for 35 minutes. The control set of cultures experienced continued anaerobic incubation.

#### Catalase assays

Overnight cultures were diluted to a final OD of 0.1 (A_600_) in fresh TSB and cultured to an OD of 8 (A_600_), at the desired HVR. Cell lysis and clarification of the supernatant were as described earlier [47]. The cell-free lysate was further diluted 50-fold and catalase activity was assayed by the addition of 5 μL of the diluted extract to 975 μL of assay buffer A (50 mM Tris, pH 8.0, 10 mM MgCl_2_, and 18 mM H_2_O_2_). The decomposition of H_2_O_2_ was monitored spectrophotometrically, as described elsewhere [49].

#### Superoxide dismutase assays

Cells were cultured, harvested, and cell pellets obtained as described above for aconitase assays or as specified in the figure legend. SOD activity in the cell lysates was determined using the xanthine oxidase-cytochrome c method as described elsewhere [50].

#### Succinate dehydrogenase assays

Cells were cultured, harvested, and cell pellets obtained as described above for aconitase assays or as specified in the figure legend. Sdh activity was determined as described previously [46]. The reaction mixture contained PBS (pH 7.5), 2 mM KCN, 742 nM XTT, 19.5 nM phenazine methosulfate and 130 mM sodium succinate (pH 7.1). The reaction was initiated by the addition of cell-free extract and reduction of XTT was monitored at 490 nm.

For all data examining enzymatic activity that is presented in this study, activity was standardized with respect to the protein concentration of the cell-free lysate and subsequent normalizations were as mentioned in the individual panels.

### Protein purification

SrrA containing a C-terminal poly-histidine affinity tag was overproduced in and purified from *E*. *coli*. *E*.*coli* BL21-AI* cells containing the pET20b-*srrA* plasmid were grown to an optical density of 0.6 (A_600_) before induction by the addition of 0.3% arabinose. Cells were harvested by centrifugation and flash frozen in liquid nitrogen. Cells were lysed by resuspension in 50 mL of Buffer A (50 mM Tris, pH 7.5, 150 mM NaCl and 5% glycerol) and passage through a chilled French pressure cell. Lysates were subsequently clarified by centrifugation and loaded onto a column of NI-NTA resin (Qiagen), equilibrated with Buffer A. The column was washed with 25 column volumes of wash buffer (50 mM Tris, pH 7.5, 2 M NaCl and 5% glycerol) before equilibration with 5 column volumes of Buffer A. Protein was eluted using a linear gradient with Buffer A containing 200 mM Imidazole. Fractions were collected and analyzed by SDS-PAGE. Fractions containing SrrA protein were pooled and dialyzed overnight at 4°C in Buffer B (50 mM Tris, pH 7.5, 110 mM KCl, 5% glycerol and 1 mM DTT). Dialyzed protein was loaded onto a column containing Q-Sepharose resin pre-equilibrated with Buffer B. The column was washed with Buffer B and SrrA was eluted using a linear gradient of Buffer B containing 2 M KCl. Fractions were collected and analyzed using SDS-PAGE. Fractions containing SrrA protein at >95% purity were pooled and dialyzed overnight against Buffer B containing 0.01 M EDTA. SrrA was concentrated over a 3,000 Dalton molecular-mass-cutoff membrane (Amicon YM-3). Purified protein was further verified using western blots as previously described [51]. Concentrated protein was flash frozen in single use aliquots in liquid nitrogen. Concentration for proteins was routinely determined using micro-Biuret assay [52].

### DNA mobility shift assays

Primers were used amplify 150 base pairs upstream of the annotated translational start sites of the *rpsC*, *srrA*, and *dps* genes using PCR. A biotin labeled primer was used to create all amplicons except those used as cold competitor controls. All amplicons were purified using a 3.5% NATIVE-PAGE gel, as described elsewhere (National Diagnostics). Prior to binding assays, phosphorylated SrrA (Srr~P) was obtained by incubating SrrA with 90 mM acetylphosphate at room temperature for 100 minutes. Binding assays were carried out in a reaction volume of 27 μL containing 15–146 ng of SrrA protein, 8 fmol of labeled DNA, 30 ng non-specific poly(dI-dC) DNA, 20 mM EDTA and 5 mM MgSO_4_. After 20 minutes of incubation at room temperature the binding reactions were subjected to electrophoresis on a 3.5% TBE gel. The DNAs were subsequently transferred to a Hybond N membrane (GE Healthcare) and the blots were developed using chemiluminescent detection (Lightshift Kit, Pierce). Specific DNA::protein interactions were verified using 200-fold molar excess of a cold competitor control.

## Results

### A strain lacking the staphylococcal respiratory regulatory system (SrrAB) has increased susceptibility to H_2_O_2_

**Fig 1:**
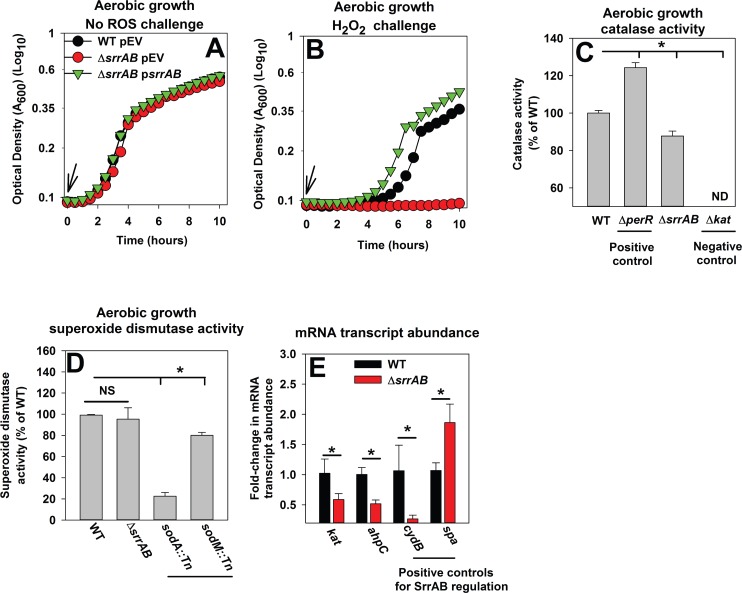
A Δ*srrAB* strain is deficient in growth upon H_2_O_2_ challenge and has decreased transcription of *ahpC* and *kat* during aerobic growth. Panels A-B; A Δ*srrAB* strain is deficient in growth upon H_2_O_2_ challenge. The WT (JMB1100) with pCM28 empty vector (p*EV*) and the Δ*srrAB* strain (JMB1467) with pCM28 (p*EV*) or p*srrAB* were diluted into defined medium to a final optical density of 0.025 (A_600_) and challenged with vehicle control (Panel A) or 1.32 mM hydrogen peroxide (H_2_O_2_) (Panel B) at the point of inoculation (indicated by arrow). Panel C; Catalase (Kat) activity is decreased in a Δ*srrAB* strain. Kat activity was assessed in cell-free lysates from the WT, Δ*srrAB*, Δ*perR* (JMB2151), and Δ*kat* (JMB2078) strains. ND represents no detectable activity. Panel D; Superoxide dismutase (Sod) activity is not decreased in a Δ*srrAB* strain. Sod activity was assessed in cell-free lysates from the WT, Δ*srrAB*, *sodA*::*Tn* (JMB 6326), and *sodM*::*Tn* (JMB 2968) strains. Panel E; The abundances of mRNA transcripts corresponding to genes that encode for H_2_O_2_ detoxification proteins are lower in the Δ*srrAB* strain. The abundances of the *ahpC*, *kat*, *cydB* and *spa* transcripts were determined in the WT and Δ*srrAB* strains. The data were normalized to 16s rRNA transcript levels and are presented as fold-change relative to the WT. Representative data are shown in Panels A-B and experiments were repeated on three independent occasions. Data in Panel C-E represent the average of biological triplicates with standard deviations shown. Where indicated, two-tail student t-tests were performed on the data and * represents statistically significant data with *P*< 0.05.

We screened a library of *S*. *aureus* strains lacking individual two-component regulatory systems (TCRS) for H_2_O_2_ sensitivity. A strain lacking the SrrAB TCRS had an extended lag-time before outgrowth when compared to the parental USA300_LAC strain (wild-type; WT) when challenged with H_2_O_2_ (Fig 1A and 1B). Returning *srrAB in trans* negated the H_2_O_2_ sensitivity of the Δ*srrAB* strain verifying that the absence of SrrAB was resulting in the sensitivity phenotype (Fig 1A and 1B). The time necessary for the WT to initiate outgrowth increased in synchrony with increasing concentrations of H_2_O_2_ (S1A Fig). The lag-times for the Δ*srrAB* strain also increased in synchrony with increasing H_2_O_2_, but the lag-times were significantly greater than those of the WT (S1B Fig). However, challenge with H_2_O_2_ did not significantly alter the generation times of either strain (data not shown).

To further examine this phenomenon, cells were resuspended in buffer and challenged with a bolus of H_2_O_2_. The stress was terminated by the addition of catalase and the viable cells were quantified. The Δ*srrAB* strain had a ~10-fold decrease in survival post H_2_O_2_ challenge (S1C Fig) and this phenotype could be genetically complemented. Moreover, increasing the initial inoculum of the Δ*srrAB* strain 10-fold resulted in a lag-time before outgrowth that was similar to that of the WT upon H_2_O_2_ challenge (data not shown). We concluded that the increased lag-time of the Δ*srrAB* strain upon H_2_O_2_ challenge was likely an outcome of decreased cell viability.

### SrrAB positively influences basal expression of H_2_O_2_ resistance factors

Ahp and Kat detoxify H_2_O_2_ and SodA has been proposed to facilitate resistance to H_2_O_2_ in alternate bacteria [16, 53–56]. PerR represses expression of peroxide resistance factors including Kat [57]. Catalase (Kat) activity was measured in cell-free lysates harvested from the WT, Δ*srrAB*, Δ*kat* (negative control) and Δ*perR* (positive control) strains, cultured aerobically. The Δ*srrAB* strain displayed ~15% lower catalase activity than the WT (*P*<0.05) (Fig 1C). Kat activity was increased by ~25% in the Δ*perR* mutant while no activity was detected in the Δ*kat* strain. Superoxide dismutase activity was measured in cell-free lysates harvested from the WT, Δ*srrAB*, *sodA*::*Tn*, and *sodM*::*Tn* strains cultured aerobically. Sod activity was not decreased in the Δ*srrAB* strain, but it was decreased by ~75% in the *sodA*::*Tn* strain and by ~20% in the *sodM*::*Tn* strain (Fig 1D).

We next examined whether SrrAB modulated the transcript levels of *ahpC* and *kat*. SrrAB is known to alter transcription of *spa* and *cydB* and SrrA directly modulates transcription of *spa*. The *spa* and *cydB* genes are repressed and activated by SrrAB, respectively [31, 34, 35]. The abundances of the transcripts corresponding to *ahpC*, *kat*, and *cydB* were reduced in the Δ*srrAB* strain (~2–3 fold for each gene) (*P*<0.05) (Fig 1E). The abundance of the *spa* transcript was increased in the Δ*srrAB* strain (~2 fold) (*P*<0.05) (Fig 1E).

We assessed whether SrrAB was required for the induction of *ahpC* and *kat* in cells challenged with H_2_O_2_. The WT and Δ*srrAB* strains were cultured to post-exponential growth phase, challenged with a bolus of H_2_O_2_, and the transcript abundances were assessed. The changes in transcript levels for *ahpC* and *kat*, upon H_2_O_2_ challenge, were indistinguishable between the WT and Δ*srrAB* strains. (S2 Fig).

From Fig 1, S1 Fig and S2 Fig, we concluded that SrrAB a) positively influences the basal transcript levels for *ahpC* and *kat*, and b) manages the mutually inclusive regulation of genes required for H_2_O_2_ resistance, aerobic respiration (*cydB*) and virulence (*spa*) during aerobic growth.

### SrrAB positively influences Dps expression and iron chelation or introduction of a null *perR* allele alleviates the deficient survival of the Δ*srrAB* strain upon H_2_O_2_ challenge

**Fig 2:**
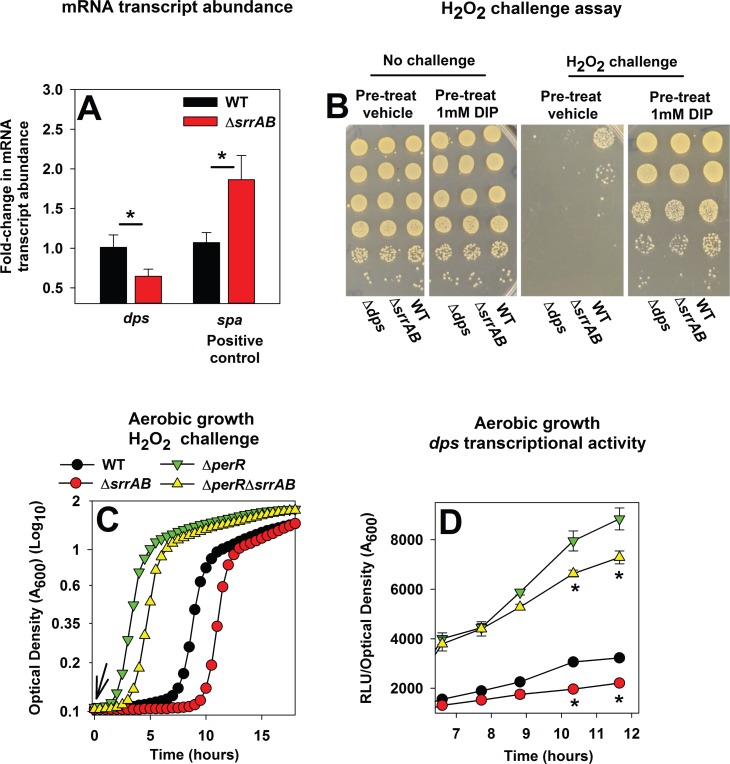
SrrAB positively influences Dps expression and iron chelation or introduction of a Δ*perR* allele alleviates the deficient survival of a Δ*srrAB* mutant upon H_2_O_2_ challenge. Panel A; The abundance of the *dps* transcript is lower in the Δ*srrAB* strain. The abundances of the *dps* and *spa* transcripts were determined in the WT (JMB1100) and Δ*srrAB* (JMB1467) strains from the cDNA libraries described in Fig 1E. The data were normalized to 16s rRNA transcript levels and are presented as fold-change relative to the WT. Panel B; Pre-incubation of the Δ*srrAB* and Δ*dps* strains with a metal chelator alleviates their sensitivity towards H_2_O_2_ challenge. The WT, Δ*srrAB*, and Δ*dps* (JMB2092) strains were cultured aerobically and subsequently incubated in buffer with vehicle control or 1 mM 2,2 dipyrydyl. Cells were then challenged with H_2_O_2_ and survival was determining colony-forming units (CFU). Panel C; Introduction of a Δ*perR* allele mitigates the H_2_O_2_ sensitivity phenotype of the Δ*srrAB* strain. The WT, Δ*srrAB*, Δ*perR* (JMB2151), and Δ*perR* Δ*srrAB* (JMB2615) strains were diluted into TSB and challenged with 1.57 mM H_2_O_2_ at the point of inoculation (indicated by arrow). Panel D; SrrAB and PerR influence *dps* transcriptional activity independent of one another. The transcriptional activity of *dps* was assessed in the WT, Δ*srrAB*, Δ*perR* and Δ*perR* Δ*srrAB* strains containing *gfp* under the transcriptional control of the *dps* promoter (pCM11_*dps*). Representative data are displayed in Panels B and C and experiments were performed on least three independent occasions. Data in Panels A and D represent the average of biological triplicates with standard deviations shown. Two-tail student t-tests were performed on the data in Panel A and * represents statistically significant data with *P*< 0.05.

Dps protects cells from H_2_O_2_ toxicity [21–23]. SrrAB could positively influence *dps* transcription, and thereby influence H_2_O_2_ resistance. Consistent with this theory, the abundance of the *dps* transcript was decreased in the Δ*srrAB* strain (Fig 2A). In part, Dps imparts cellular protection by binding and sequestering free Fe, and thereby suppressing Fenton chemistry. We examined whether pre-incubation of the Δ*srrAB* and Δ*dps* strains with an iron chelator would mitigate the survival of these strains upon H_2_O_2_ challenge. The Δ*srrAB* and Δ*dps* strains that had been pre-incubated with vehicle-control, followed by H_2_O_2_ challenge, displayed decreased survival when compared to the WT. However, pre-incubation of strains with the cell permeable metal chelator 2,2-dipyrydyl, followed by H_2_O_2_ challenge, rescued the phenotypes of the Δ*srrAB* and Δ*dps* strains (Fig 2B).

Next, we examined whether increasing the expression of H_2_O_2_ resistance factors in the Δ*srrAB* strain would mitigate the H_2_O_2_ sensitivity phenotype of this strain. PerR represses transcription of *ahpC*, *kat*, and *dps* (52). As expected, the Δ*perR* strain displayed increased resistance to H_2_O_2_ challenge (Fig 2C). Introduction of the Δ*perR* mutation mitigated the H_2_O_2_ sensitivity phenotype of the Δ*srrAB* strain. The Δ*perR* Δ*srrAB* strain displayed increased resistance towards H_2_O_2_ relative to the WT; however, this resistance was lower than that of the Δ*perR* mutant. These data suggested that PerR and SrrAB influence the expression of peroxide resistance factors independent of one another. Consistent with this premise, Kat activity was lower in the Δ*perR* Δ*srrAB* strain than in the Δ*perR* strain (S3 Fig). Moreover, the transcriptional activity of *dps* was lower in the Δ*perR* Δ*srrAB* strain than the Δ*perR* strain (Fig 2D).

From the data in Fig 2 and S3 Fig we concluded that a) the H_2_O_2_ sensitivity of the Δ*srrAB* strain was the result of decreased expression of H_2_O_2_ resistance factors, and b) SrrAB influences H_2_O_2_ resistance factors independently of PerR.

### SrrA binds to the promoter regions of *dps* and *srrA*

SrrA is an OmpR family response regulator that binds to the *srrA* promoter [31]. OmpR type regulators may bind inverted repeat sequences and RR binding regions can have an internal spacer of a varying length and sequence [58–62]. Visual inspection of the *srrA* promoter revealed the presence of an inverted repeat sequence separated by a four base-pair spacer region (AAATAN_4_TTTAT). The potential binding site was also found in the promoter regions of *icaA* (SAUSA300_2600), *tsst* (SA1819), and *agrB* (P2 promoter, SAUSA300_1989) (S4 Fig); each of which is a direct binding target for SrrA~P [31, 35]. The inverted repeat was not found to occur in the promoter regions for four randomly selected genes in LAC (SAUSA300- 2112, 1112, 0124 and 2198). Allowing for a maximum of one mismatch, the inverted repeat was found to be present in the promoter region for *dps* (S4 Fig). A putative SrrA binding sequence was not observed in the promoter regions for *kat* or *ahpC*.

**Fig 3:**
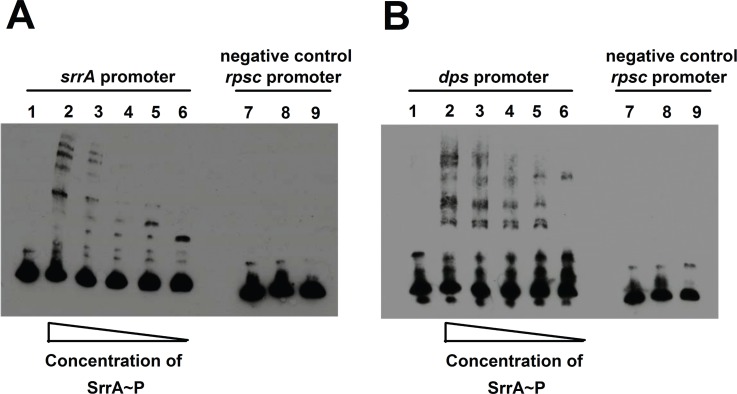
SrrA binds to DNA fragments immediately preceding the annotated transcriptional start sites for *srrA* and *dps*. Panels A and B; Electromobility gel shift assays (EMSAs) demonstrating binding of SrrA to DNAs that correspond to the 150 base pair segments immediately preceding the annotated transcriptional start sites for the *srrA* (A) and *dps* (B). EMSAs were performed with SrrA (15–146 ng) and 8 fM of biotin labeled DNA. For each gel, the samples in lane 1 contain 146 ng SrrA with labeled sample DNA and a 125-fold excess of non-labeled (cold) competitor DNA. The samples in lanes 2–5 contain labeled DNA with varying amounts of SrrA protein (15–146 ng). The samples in lane 6 contain labeled DNA, but no SrrA. The samples in lanes 7–9 show that the interaction of SrrA with DNA is specific. The samples in lane 7 contain 146 ng SrrA with *rpsC* promoter DNA and a 125-fold excess of non-labeled (cold) competitor DNA. The samples in lanes 8 contain *rpsC* promoter DNA with 146 ng of SrrA. The samples in lane 9 contain *rpsC* promoter DNA, but no SrrA.

Electromobility gel shift assays (EMSAs) were used to assess whether SrrA is capable of binding to the promoter region of *dps*. Binding to the *srrA* promoter was assessed as a positive control. The promoter of *rpsC* lacks the putative SrrA binding region, and was included as a negative control for non-specific DNA binding. SrrA bound to 150 base pair DNA fragments corresponding to the sequences located upstream of the annotated transcriptional start sites for *srrA* and *dps* in a concentration dependent manner (Fig 3A and 3B). SrrA did not bind to the *rpsC* promoter at the highest protein concentrations examined (Fig 3A and 3B). Consistent with the SrrA::DNA interactions being specific, the addition of excess unlabeled promoter DNA decreased the amount of biotin labeled DNA bound by SrrA. The data presented in Fig 2 and Fig 3 suggested that SrrAB directly modulates the transcription of *dps*.

### SrrAB influences H_2_O_2_ resistance independent of CidA and CidB in *S*. *aureus* LAC

While this manuscript was under preparation, a study was published investigating the influence of SrrAB upon programmed cell death mediated by *cidABC* [63]. The study used a special medium (TSB medium amended with 35 mM glucose) that induces cell death phenotypes [63]. The authors found that SrrAB represses the transcription of *cidABC* when cultured in the cell death media. They also found that a Δ*srrAB* mutant was sensitive to H_2_O_2_ intoxication and this phenotype was mitigated by the introduction of a null *cidA* or a null *cidB* allele. These data led to the hypothesis that the H_2_O_2_ sensitivity of the Δ*srrAB* strain was the result of increased CidA and CidB activity. However, we found that the introduction of a *cidA*::*Tn* or a *cidB*::*Tn* into the LAC Δ*srrAB* strain did not alleviate H_2_O_2_ sensitivity when cultured in TSB medium (S5 Fig). Strains lacking Kat or PerR displayed increased sensitivity and resistance phenotypes towards H_2_O_2_, as expected (S5 Fig). We concluded that the H_2_O_2_ sensitivity of the *S*. *aureus* LAC Δ*srrAB* strain cultured under our growth conditions and in TSB medium is independent of CidA or CidB.

### SrrAB negatively influences *ahpC* and *kat* transcription during early exponential growth in TSB and during fermentative growth

**Fig 4:**
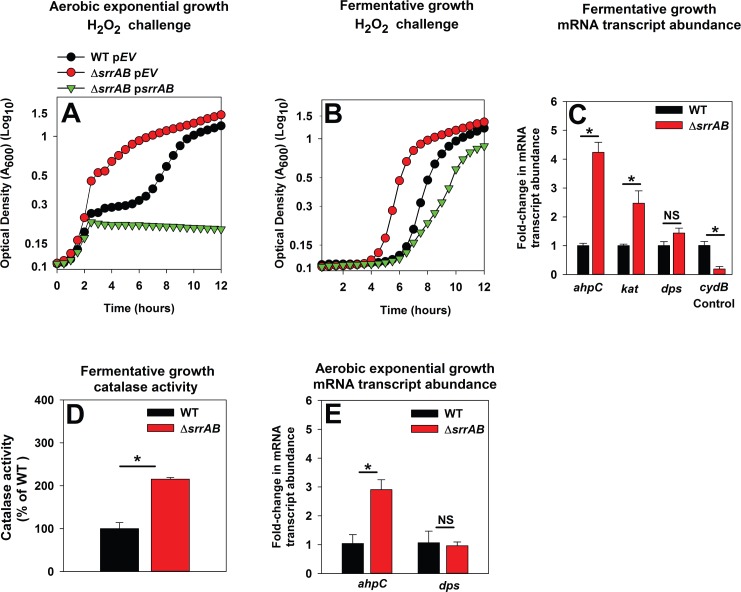
SrrAB negatively influences *ahpC* and *kat* during the early exponential growth and fermentative growth phases upon culture in TSB medium. Panels A-B; The Δ*srrAB* strain is resistant towards H_2_O_2_ challenge when cultured aerobically to exponential growth (Panel A) or fermentatively (Panel B). The WT (JMB1100) with pCM28 empty vector (p*EV*) and the Δ*srrAB* strain (JMB1467) with pCM28 (p*EV*) or p*srrAB* were cultured in TSB aerobically to exponential growth phase (2 doublings) (Panel A) or fermentatively (Panel B). The cells were subsequently challenged with 2.6 mM (Panel A) or 0.22 mM H_2_O_2_ (Panel B) and growth was monitored aerobically. Panels C; The mRNA transcript abundances corresponding to *ahpC* and *kat* are increased in the Δ*srrAB* strain cultured fermentatively. The abundances of the *ahpC*, *kat*, *dps*, and *cydB* mRNA transcripts were determined in the WT and Δ*srrAB* strains cultured fermentatively. Panel D; Catalase (Kat) activity is increased in a Δ*srrAB* strain cultured fermentatively. Kat activity was assessed in cell-free lysates from the WT and Δ*srrAB* strains after fermentative culture. Panel E; The abundance of the *ahpC* transcript is increased in the Δ*srrAB* strain cultured aerobically to exponential growth. Transcript abundances corresponding to *ahpC* and *dps* were quantified in the WT and Δ*srrAB* strains cultured aerobically to exponential growth phase. The data in Panels C and E were normalized to 16s rRNA transcript levels and are presented as fold-change relative to the WT. Data shown in panels C-E represent the average of biological triplicates with standard deviations shown. Representative growth profiles are presented in Panels A and B and experiments were performed on least three independent occasions. Where indicated, two-tail student t-tests were performed on data and * denotes p< 0.05 and NS denotes not significant.

While we were revising this manuscript, Oogai *et al*. published a report finding that during aerobic exponential-growth in TSB medium a *S*. *aureus* Δ*srrAB* mutant in the MW2 background had increased transcription of *ahpC* and *kat* (but not *dps*), as well as increased resistance towards H_2_O_2_ [64]. In contrast to Oogai *et al*., the H_2_O_2_ sensitivity phenotypes reported herein and by Windham *et al*. were conducted upon cells cultured to post-exponential growth. We tested the hypothesis that SrrAB negatively modulates H_2_O_2_ resistance during exponential growth. Consistent with our reasoning, a LAC Δ*srrAB* strain, cultured to exponential phase, followed by challenge with H_2_O_2_, displayed increased resistance and this phenotype could be genetically complemented (Fig 4A).

When *S*. *aureus* is sub-cultured into TSB medium the TCA cycle is largely repressed during the initial period of growth (exponential growth) and fermentative by-products accumulate in the extracellular milleu [65, 66]. Subsequently, TCA cycle function increases as the fermentative byproducts produced during initial growth are utilized to support respiratory growth (post-exponential growth) [42, 65, 66]. Decreased TCA cycle activity during exponential growth would result in reduced flux through respiratory pathways. We examined whether SrrAB modulates H_2_O_2_ resistance negatively during fermentative growth. Strains were cultured anaerobically in the absence of a terminal electron acceptor (fermentative growth), followed by challenge with H_2_O_2_. Subsequent growth was monitored aerobically. The Δ*srrAB* strain displayed greater resistance to H_2_O_2_ challenge and this phenotype could be genetically complemented (Fig 4B). The growth of the non-challenged Δ*srrAB* and WT strains was indistinguible (data not shown).

Transcriptional analyses supported the H_2_O_2_ resistance phenotypes observed during fermentative and aerobic exponential growth. The transcript levels for *kat* and *ahpC* were increased ~5- and ~3-fold in the Δ*srrAB* strain during fermentative growth (P<0.05), while those for *dps* were not significantly altered (Fig 4C). Moreover, Kat activity in cell-free extracts of the fermentatively cultured Δ*srrAB* strain was ~200% higher (Fig 4D). Likewise, transcript levels for *ahpC* were increased ~3 fold in the Δ*srrAB* strain during aerobic exponential growth, while those for *dps* were not significantly altered (Fig 4E). These findings led us to conclude that SrrAB negatively influences expression of AhpC and Kat during growth conditions when respiratory activity is low or absent.

### A Δ*srrAB* mutant incurs increased damage to aconitase during post-exponential aerobic growth

Aconitase (AcnA) requires a solvent exposed [Fe_4_-S_4_] cluster for function [48]. Enzymes with solvent exposed FeS cofactors are poisoned by H_2_O_2_ [2–5]. We tested the hypothesis that SrrAB manages the expression of genes required for H_2_O_2_ resistance during respiratory growth, and thereby imparts protection to AcnA from ROS-induced damage.

**Fig 5:**
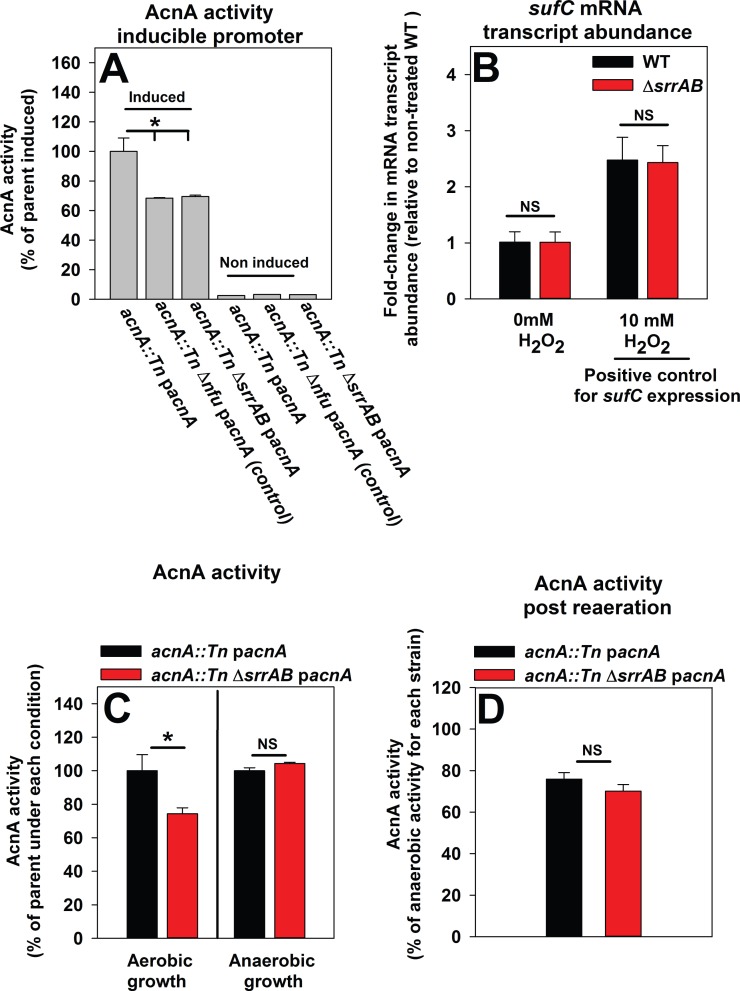
A Δ*srrAB* strain incurs increased damage to aconitase when cultured aerobically. Panel A; The activity of aconitase (AcnA) is decreased in a Δ*srrAB* mutant and this phenotype is independent of *acnA* transcription levels. AcnA activity was assessed in the *acnA*::*Tn* (JMB 3537; parent), *acnA*::*Tn* Δ*nfu* (JMB 3538), and *acnA*::*Tn* Δ*srrAB* (JMB 4367) strains carrying p*acnA*, which contains *acnA* under the transcriptional control of a xylose inducible promoter. Strains were cultured aerobically in the presence (induced) or absence (not induced) of 1% xylose. Panel B; The *sufC* mRNA transcript accumulates to similar levels in the WT and Δ*srrAB* strains. The WT (JMB1100) and Δ*srrAB* (JMB1467) strains were cultured aerobically and challenged with either 10 mM H_2_O_2_ or vehicle control and the abundance of the *sufC* mRNA transcript was quantified. Data were normalized to the 16s rRNA transcript levels and are presented as fold-change relative to the WT strain. Panel C; The activity of AcnA is similar in the WT and Δ*srrAB* strains when cultured anaerobically. AcnA activity was assessed in the *acnA*::*Tn* (JMB 3537; parent) and *acnA*::*Tn* Δ*srrAB* (JMB 4367) strains carrying p*acnA* that were cultured either aerobically or anaerobically. Panel D; A Δ*srrAB* mutant does not display increased dioxygen damage to AcnA. The *acnA*::*Tn* (JMB 3537; parent) and *acnA*::*Tn* Δ*srrAB* (JMB 4367) strains carrying p*acnA* were cultured anaerobically for 4.5 hours, treated with a protein synthesis inhibitor (100 μg mL^-1^ rifampicin) and either exposed to dioxygen or incubated anaerobically for 35 minutes subsequent to determining the activity of AcnA. Data in Panels A-D represent the average of biological triplicates. Where indicated, two-tail student t-tests were performed on data and * denotes p< 0.05 and NS denotes not significant.

We constructed *acnA*::*Tn* and *acnA*::*Tn* Δ*srrAB* strains and returned *acnA* into the strains under the transcriptional control of a xylose inducible promoter (p*acnA*). Introduction of p*acnA* allows for the control of *acnA* transcription, thereby negating potential changes in *acnA* transcription between strains. Nfu is necessary for the maturation of holo-AcnA; therefore, an *acnA*::*Tn* Δ*nfu* strain carrying p*acnA* was included as a positive control [47]. AcnA activity was assessed during post-exponential growth and found to be ~25% lower in the Δ*nfu* and Δ*srrAB* strains (Fig 5A).

Altered transcription of genes required for the synthesis of FeS clusters could result in decreased AcnA activity in the Δ*srrAB* strain. The *sufCDSUB* gene products are responsible for the synthesis of FeS clusters and decreased transcription of *sufCDSUB* results in decreased FeS enzyme activity (Roberts C., *et al*., in revision). Transcription of *sufC* was not altered in the Δ*srrAB* strain (Fig 3B). Transcription of *sufC* increases upon H_2_O_2_ challenge [47]. However, transcription of *sufC* was induced to a similar extent (~2 fold) in both the WT and Δ*srrAB* strains upon H_2_O_2_ challenge (Fig 5B) [47]. Likewise, *suf* transcriptional activity was similar in the WT and Δ*srrAB* strains over time (S6 Fig).

Two experiments were conducted to determine whether increased damage by dioxygen underlies the low AcnA activity observed in the Δ*srrAB* mutant. First, AcnA activity was assessed in the *acnA*::*Tn* and *acnA*::*Tn* Δ*srrAB* strains carrying p*acnA* and cultured in the presence and absence of dioxygen. Culturing the Δ*srrAB* mutant anaerobically restored AcnA activity (Fig 5C). Second, the same strains were cultured anaerobically, treated with a protein synthesis inhibitor (rifampicin), and one-half of the cultures were exposed to dioxygen [8], while the remainder were incubated anaerobically. Exposure of cells to dioxygen resulted in a decrease in AcnA activity, but importantly, the activity was indistinguishable between the two strains (Fig 5D). From Fig 5 we concluded that the Δ*srrAB* mutant had decreased AcnA activity when cultured aerobically and this phenotype was a) not an outcome of decreased transcription of *sufC*, or b) increased damage by dioxygen.

### SrrAB increases the ability of *S*. *aureus* to resist H_2_O_2_ intoxication during periods of high respiratory flux protecting AcnA

Respiratory flux is subject to the concentration of the terminal electron acceptor in the culture medium and SrrAB is responsive to changes in respiratory status [36, 66, 67]. SrrAB did not positively influence H_2_O_2_ resistance factors during fermentative growth and AcnA did not incur damage in a Δ*srrAB* mutant following fermentative growth. Thus, we hypothesized that the influence of SrrAB upon the expression of H_2_O_2_ resistance factors, and the subsequent protection imparted to AcnA, would be altered with respect to dioxygen concentrations in the medium and the ensuing changes in respiratory flux.

Dioxygen diffusion into growth media is a function of the aeration experienced by cultures. Aeration of batch cultures can be modified by altering the culture vessel headspace to the medium volume ratio (HVR). Decreased HVR leads to decreased aeration and less dissolved dioxygen in the medium [66–69]. Culturing *S*. *aureus* under low aeration (HVR 1.25) results in a metabolic block at the TCA cycle [66], which would result in reduced flux through respiratory pathways.

**Fig 6:**
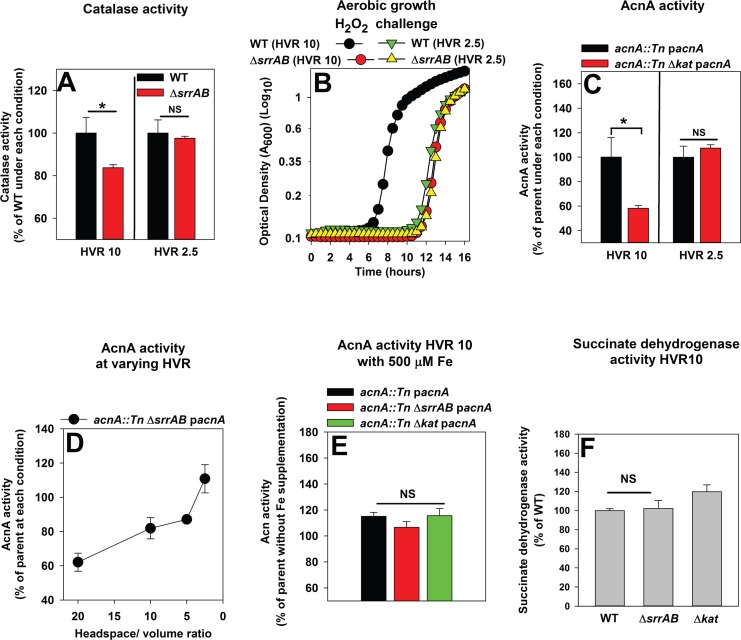
SrrAB upregulates H_2_O_2_ resistance during periods of high aeration and imparts protection to AcnA. Panel A; SrrAB positively influences catalase (Kat) expression in cells cultured under high aeration, but not under low aeration. Kat activity was assessed in cell-free lysates generated from the WT (JMB1100) and Δ*srrAB* (JMB1467) strains cultured at a culture vessel headspace to liquid volume ratios (HVR) of 2.5 or 10. Panel B; SrrAB increases the ability of *S*. *aureus* to withstand H_2_O_2_ intoxication when cells are cultured under high aeration. The WT and Δ*srrAB* strains were cultured at a HVR of 10 or 2.5, diluted into fresh TSB medium and challenged with 1.57 mM H_2_O_2_ at the point of inoculation. Panel C; AcnA activity is decreased in a strain lacking catalase when cultured under high aeration, but not under low aeration. AcnA activity was assessed in cell-free lysates harvested from the *acnA*::*Tn* (JMB3537; parent) and the *acnA*::*Tn* Δ*kat* (JMB7107) strains carrying p*acnA* cultured at a HVR of 2.5 or 10. Panel D; AcnA activity is decreased in a Δ*srrAB* mutant cultured under high aeration, but not when cultured under low aeration. AcnA activity was assessed in cell-free lysates from the *acnA*::*Tn* (JMB3537; parent) and *acnA*::*Tn* Δ*srrAB* (JMB4367) strains carrying p*acnA* cultured at various HVRs. Panel E; The decreased AcnA activity under high aeration in strains defective in H_2_O_2_ resistance is restored by supplementation of growth medium with Fe. AcnA activity was assessed in cell-free lysates harvested from the *acnA*::*Tn* (JMB3537; parent), *acnA*::*Tn* Δ*srrAB* (JMB4367), and *acnA*::*Tn* Δ*kat* (JMB7107) strains carrying p*acnA*. Cells were cultured at HVR of 10 and in the presence or absence of 500 μM Fe. Panel F; Succinate dehydrogenase (Sdh) activity is not decreased in strains deficient in H_2_O_2_ resistance. Sdh activity was assessed in cell-free lysates from the WT, Δ*srrAB*, and Δ*kat* (JMB2078) strains cultured at a HVR of 10. Data shown in Panels A, C and D-F represent the average of biological triplicates with standard deviations shown. Representative growth profiles are presented in Panel B and experiments were performed on least three independent occasions. Where indicated, two-tail student t-tests were performed on data and * denotes p< 0.05 and NS denotes not significant.

We examined whether SrrAB alters the expression of H_2_O_2_ resistance factors in response to culture aeration. Kat activity was reduced by ~20% in the Δ*srrAB* strain when cultured under high aeration (HVR 10), but was statistically indistinguishable from that of the WT when cultured under low aeration (HVR 2.5) (Fig 6A). Increased expression of H_2_O_2_ resistance factors would be predicted to result in greater resistance towards H_2_O_2_ in cells cultured with high aeration. Consistent with this idea, WT cells cultured under high aeration (HVR 10) were more resistant to H_2_O_2_ challenge than cells cultured under low aeration (HVR 2.5). In contrast, the sensitivity of the Δ*srrAB* strain towards H_2_O_2_ was not altered as a variable of HV ratio (Fig 6B).

We reasoned that a strain deficient in scavenging H_2_O_2_ would have increased AcnA damage resulting in decreased AcnA activity when cultured under high aeration (HVR 10). The *acnA*::*Tn* Δ*kat* strain carrying p*acnA* displayed lower AcnA activity than the *acnA*::*Tn* strain carrying p*acnA* when cultured under high aeration (HVR 10), but not when cultured under low aeration (HVR 2.5) (Fig 6C). AcnA activity was also assessed in the *acnA*::*Tn* and *acnA*::*Tn* Δ*srrAB* strains carrying p*acnA* following culture under varying HVRs. AcnA activity in the Δ*srrAB acnA*::*Tn* strain with p*acnA* was significantly lower than that of the *acnA*::*Tn* strain with p*acnA* (~60%) when cultured under high aeration (HVR 20). However, as the HVR was decreased, the difference in AcnA activity between the two strains diminished. AcnA activity was fully restored in Δ*srrAB* mutant after culture at a HVR of 2.5 (Fig 6D).

Supplementing the growth medium with excess iron (Fe) restores the activities of FeS cluster requiring dehydratase enzymes damaged by oxidation [70, 71]. The *acnA*::*Tn*, *acnA*::*Tn* Δ*srrAB*, and *acnA*:*Tn* Δ*kat* strains carrying p*acnA* were cultured under high aeration (HVR 10) with and without Fe supplementation (500 μM). Supplementation of media with Fe recovered AcnA activity in the Δ*srrAB* and Δ*kat* strains (Fig 6E).

Succinate dehydrogenase (Sdh) also requires Fe-S clusters for function. However, the Fe-S clusters of Sdh are protected by the polypeptide providing increased stability [72, 73]. Consequently, exposure of up to 1mM H_2_O_2_ does not inactivate Sdh [72, 73]. Sdh activity was not decreased in the Δ*srrAB* or Δ*kat* strains cultured under high aeration (HVR 10) (Fig 6F). These findings confirmed that a) the Δ*srrAB* and Δ*kat* strains do not have a broad deficiency in the function of FeS proteins, and b) H_2_O_2_ accumulation in *S*. *aureus* predominantly damages solvent exposed FeS clusters.

The data presented in Fig 6 demonstrated that *S*. *aureus* increased the ability to resist H_2_O_2_ when cultured under high aeration and these alterations arose due to regulatory changes mediated by SrrAB. Further, strains deficient in detoxifying endogenously produced H_2_O_2_ (Δ*srrAB* and Δ*kat*) displayed decreased AcnA activity when cultured under high aeration, but not low aeration.

### SrrAB positively influences transcription of *scdA*, which encodes for a FeS cluster repair protein

**Fig 7:**
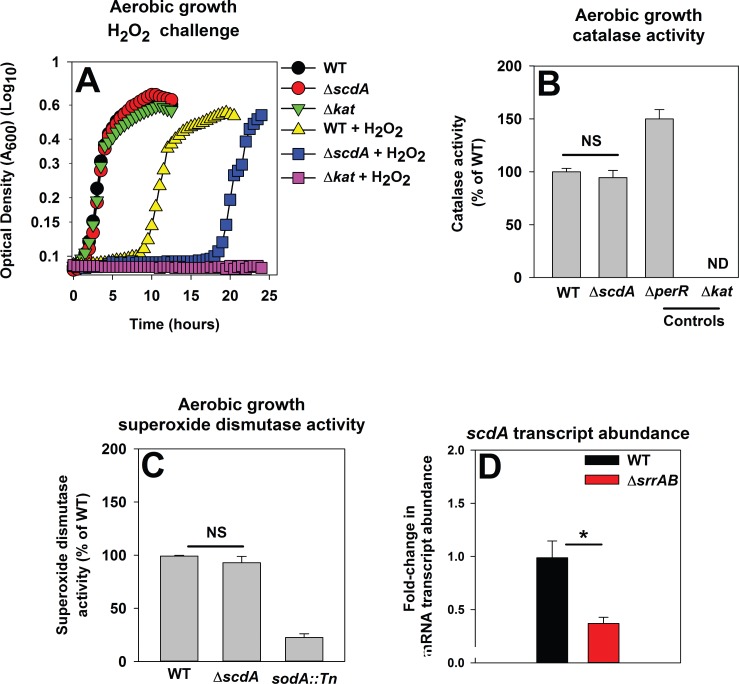
SrrAB positively influences the transcription of *scdA*. Panel A; A *S*. *aureus* USA300_LAC Δ*scdA* mutant is sensitive to H_2_O_2_ intoxication. The WT (JMB1100), Δ*scdA* (JMB1254), and Δ*kat* (JMB2078) strains were cultured aerobically, diluted into fresh medium, and challenged with 1.57 mM H_2_O_2_ at the point of inoculation. Panel B; The Δ*scdA* strain is not defective in catalase (Kat) activity. Kat activity was assessed in cell-free lysates from the WT, Δ*scdA*, Δ*perR* (JMB2151), and Δ*kat* strains cultured at a HVR of 10. Panel C; The Δ*scdA* strain does not have decreased superoxide dismutase (Sod) activity. Sod activity was assessed in cell-free lysates from the WT, Δ*scdA*, and Δ*sodA*::*Tn* (JMB6326) strains cultured at a HVR of 10. Panel D; The abundance of the *scdA* mRNA transcript is lower in the Δ*srrAB* strain during post-exponential growth. The mRNA abundance corresponding to *scdA* was determined using the same cDNA libraries as described in Fig 2A. The data were normalized to 16s rRNA transcript levels and are presented as fold-change relative to the WT strain. Representative growth profiles are presented in Panel A and experiments were performed on least three independent occasions. The data in Panels B-D represent the average of biological triplicates with standard deviations shown.

The *S*. *aureus* di-iron RIC protein ScdA (YtfE in *Escherichia coli*) has a role in the repair of FeS proteins damaged by H_2_O_2_ [24, 25]_._ Previous studies on ScdA were conducted in the *S*. *aureus* strain RN4220, which lacks Sigma B and Agr funtion, which control expression of ROS resistance genes [74, 75]. We conducted experiments to determine whether a) ScdA is required for H_2_O_2_ resistance in *S*. *aureus* LAC, b) the H_2_O_2_ sensitivity of a Δ*scdA* strain is independent of altered expression of detoxification factors, and c) SrrAB modulates *scdA* transcription. *S*. *aureus* LAC Δ*scdA* and Δ*kat* strains displayed increased sensitivity towards H_2_O_2_ (Fig 7A). However, the Δ*scdA* strain did not display decreased Kat (Fig 7B) or Sod activity (Fig 7C). The transcript level corresponding to *scdA* was decreased (~3 fold) in the Δ*srrAB* strain during post-exponential growth (Fig 7D).

### The positive influence of SrrAB upon H_2_O_2_ metabolism is conserved in diverse *S*. *aureus* isolates

**Fig 8:**
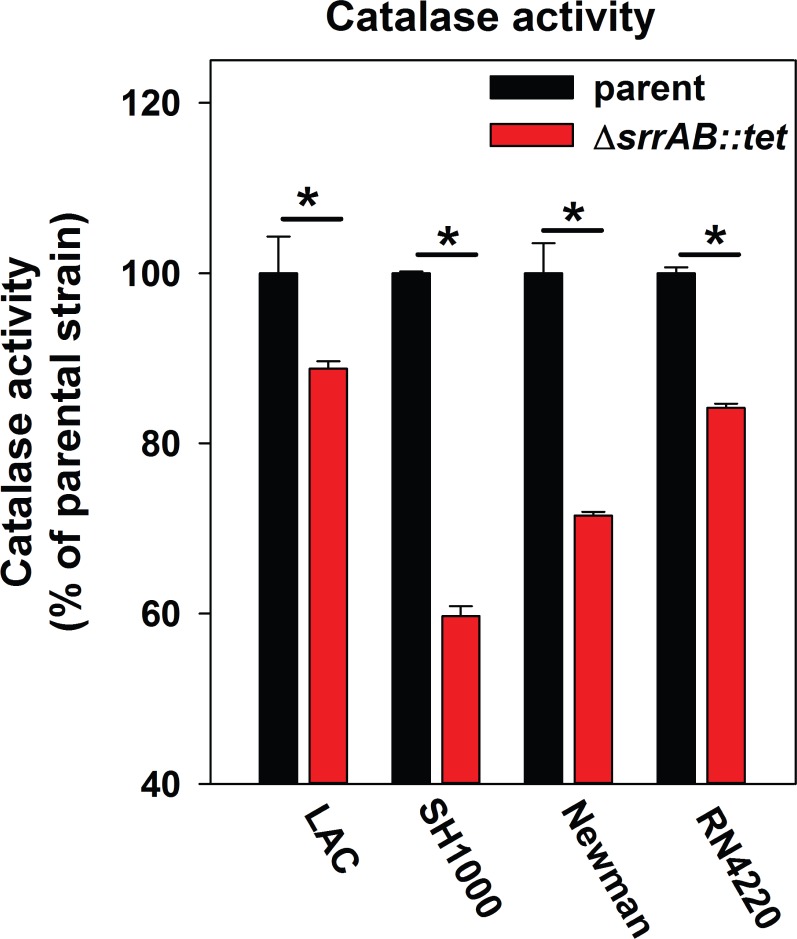
The influence of SrrAB upon aerobic respiration and H_2_O_2_ metabolism is conserved in diverse *S*. *aureus* isolates. Catalase (Kat) activity is decreased in diverse *S*. *aureus* isolates lacking SrrAB. The USA_300 LAC (JMB1100), USA300_LAC Δ*srrAB* (JMB2047), Newman (JMB1422), Newman Δ*srrAB* (JMB4751), SH1000 (JMB1323), SH1000 Δ*srrAB* (JMB4556), RN4220 (JMB1103), RN4220 Δ*srrAB* (JMB2030) strains were cultured aerobically to post-exponential growth phase and Kat activities were determined in the cell-free lysates. The data represent the average of biological triplicates with standard deviations shown.

Regulatory networks that are integral to *S*. *aureus* physiology differ between isolates [76, 77]. We examined the influence of SrrAB upon ROS metabolism by assessing catalase activity in the SH1000, Newman, and RN4220 genetic backgrounds and their isogenic Δ*srrAB* mutants. Strains lacking SrrAB displayed lower catalase activity relative to their parent strains (~10–60% lower catalase activity). When compared to a LAC background, the catalase activity was substantially lower in the Newman and SH1000 Δ*srrAB* mutants (~30% and ~60% lower, respectively) (Fig 8). The Δ*srrAB* mutants were also more sensitive than their isogenic parent strains to H_2_O_2_ challenge (data not shown).

## Discussion

The goal of this study was to further our understanding of the role of SrrAB in H_2_O_2_ resistance. During post-exponential growth a Δ*srrAB* strain was sensitive to H_2_O_2_ challenge. In *S*. *aureus*, AhpC and Kat are required for scavenging H_2_O_2_ [16]. The iron-sequestering and DNA-binding protein Dps is utilized to suppress Fenton chemistry [21–23]. ScdA (YtfE) also provides resistance to H_2_O_2_ by aiding in the repair of H_2_O_2_ damaged FeS proteins [24, 25]. The Δ*srrAB* strain has decreased transcription of *ahpC*, *kat*, *dps* and *scdA*; therefore, the H_2_O_2_ sensitivity phenotype of the Δ*srrAB* strain is likely to arise, in part, as a result of the combined effect of decreased expression of these genes. In support of this idea we found that a) increasing the expression of H_2_O_2_ resistance factors by the introduction of a null *perR* allele into the Δ*srrAB* strain increased H_2_O_2_ tolerance, and b) pre-incubation of Δ*srrAB* strain with a cell permeable iron chelator prior to H_2_O_2_ challenge, alleviated the H_2_O_2_ sensitivity phenotype. Further, purified SrrA was capable of binding to the promoter region of *dps* in EMSA assays, suggesting that SrrAB directly modulates the transcription of at least one H_2_O_2_ resistance factor.

While our manuscript was under revision, Oogai *et al*. published a study that both confirmed our findings, and yet, seemingly contradicted them [64]. Oogai *et al*. found that SrrAB negatively influences AhpC and Kat expression in the *S*. *aureus* isolate MW2 [64]. We further investigated this phenomenon in order to reconcile our findings with those of Oogai *et al*. We found that the negative influence of SrrAB upon H_2_O_2_ detoxification occurs during periods of decreased TCA cycle activity (aerobic exponential growth in TSB), which would ultimately lead to decreased flux through respiratory pathways or during fermentative growth. Moreover, SrrAB increased the ability of cells to withstand H_2_O_2_ stress when respiratory activity was high. The results presented herein confirm the findings of Oogai *et al*., as well as significantly expand upon them. The finding that SrrAB altered the H_2_O_2_ resistance of *S*. *aureus* in response to respiration are in agreement with the prevailing hypothesis that alterations in the cellular respiratory status serve as a stimulus for SrrAB ([36] and Mashruwala *et al*. in review). Our findings extend the findings of others in emphasizing the importance of careful reporting of the culture media, growth parameters, and growth phases in the study of bacterial physiology [66, 67].

It is currently unclear how SrrAB negatively influences *ahpC*, *kat* and *scdA* transcription. We did not identify putative SrrA binding sites in the promoter regions for *ahpC*, *kat*, or *scdA* suggesting SrrAB indirectly modulates their expression. Oogai *et al*. reached a similar conclusion for AhpC and Kat [64]. SrrAB, as well as it's ortholog ResDE in *Bacillus subtilis*, modulate the expression of the small non-coding regulatory RNA called RsaE/RoxS [78]. *B*. *subtilis* strains lacking RsaE/RoxS display altered expression of peroxide metabolism genes [78]. Thus, one explanation would be that SrrAB influences the expression of *kat*, *ahpC* and *ytfe* via RsaE/RoxS. We are currently testing this hypothesis. A strain lacking SrrAB also has altered respiration, redirected carbon flux, and by inference an altered redox status [36, 37]. Thus, an alternate explanation is that the physiological changes that are the result of the absence of SrrAB indirectly affect the expression of AhpC, Kat, and Ytfe.

**Fig 9:**
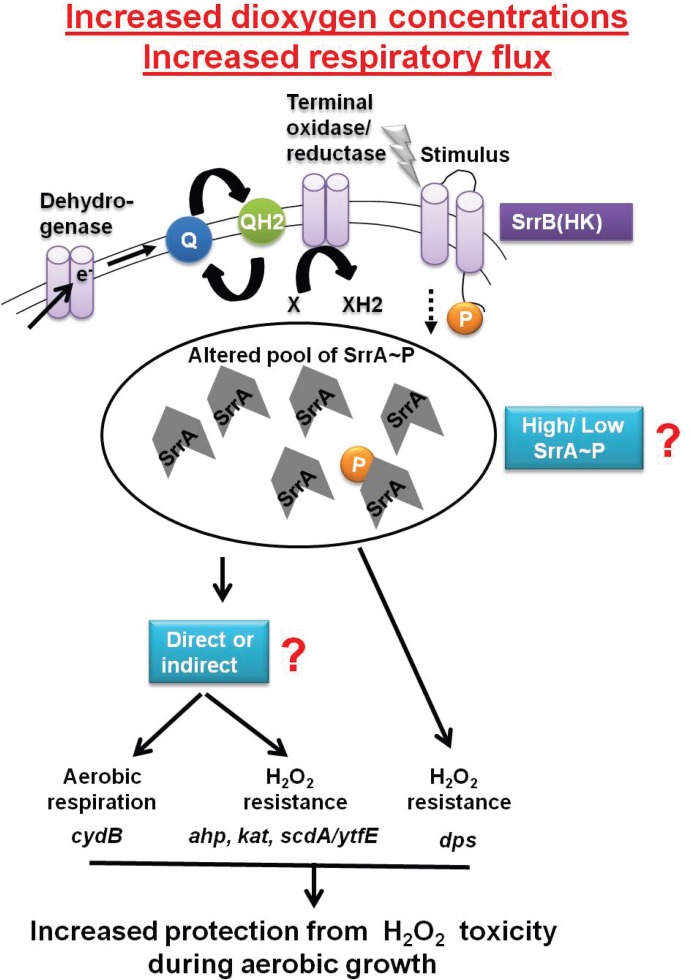
Working model for the role of SrrAB in modulating the transcription of genes utilized in H_2_O_2_ resistance and dioxygen respiration. SrrAB modulates gene transcription in response to cellular respiratory flux [36]. We propose that increased culture aeration leads to increased respiratory flux during post-exponential growth, which results in altered kinase activity of SrrB and variation in the cellular pool of SrrA~P. An altered SrrA~P pool results in increased expression of genes under the SrrAB regulon that are utilized for H_2_O_2_ resistance and dioxygen respiration. The resultant physiological changes allow for cellular homeostasis by protecting macromolecules against H_2_O_2_ toxicity that arise during dioxygen respiration.

Why would SrrAB regulate H_2_O_2_ resistance factors in *S*. *aureus*? SrrAB influences the transcription of multiple genes necessary for aerobic respiration [34] in response to respiratory flux [36]. H_2_O_2_ is a by-product of spontaneous interactions between dioxygen and components of respiratory pathways [2–5]. Therefore, H_2_O_2_ arises spontaneously during aerobic growth [2, 3]. Accumulation of as little as two μM H_2_O_2_ has been proposed to be sufficient to inhibit the growth of *E*. *coli* [79]. *S*. *aureus* cells lacking the ability to synthesize both Ahp and Kat accumulate ~25 μM H_2_O_2_. The accumulation of H_2_O_2_ is maximal during post-exponential growth, which coincides with maximal respiratory processes [16]. Consequently, during aerobic growth, when *S*. *aureus* cells are respiring, it is imperative for the cell to detoxify H_2_O_2_ and repair macromolecules damaged by H_2_O_2_. This led us to propose a working model displayed in Fig 9 wherein SrrAB manages the mutually inclusive expression of genes required for aerobic respiration, H_2_O_2_ detoxification, and the repair of damaged cellular assets (FeS clusters), thereby facilitating proficient growth.

Consistent with our model, SrrAB modulated the transcription of H_2_O_2_ detoxification factors (*kat* and *ahpC*). Protein-associated solvent accessible FeS clusters, such as the cofactor of AcnA, are among the primary cellular targets of H_2_O_2_ [7, 8, 80, 81]. A Δ*srrAB* strain had decreased AcnA activity when cultured aerobically and AcnA was restored by either a) decreasing culture aeration, which results in less dissolved dioxygen and decreased cellular respiration [66, 67], b) anaerobic growth, or c) supplementing the growth medium with Fe, which aids in the repair of oxidatively damaged FeS clusters [70, 71]. Moreover, SrrAB positively influenced the transcription of *scdA*, which functions in the repair of ROS damaged FeS clusters [24, 25]. The oxidation of FeS clusters can result in increased cytosolic free Fe, which can catalyze Fenton chemistry. Data presented suggest that SrrAB directly influences the transcription of *dps* and Dps is utilized to bind and sequester cytosolic free Fe suppressing Fenton chemistry.

Similar to SrrAB, the ArcAB system in gram-negative bacteria is responsive to the respiratory status of the cell [36, 82]. ArcAB controls the transcription of genes required for TCA cycle activity and fermentative processes. ArcAB also influences the transcription of ROS resistance genes in *E*. *coli*, *Haemophilus influenzae*, and *Salmonella enterica* [83–85]. In a direct parallel to SrrAB, ArcAB also alters the transcription of *dps*

SrrAB coregulated the transcription of genes encoding for virulence factors along with genes encoding for aerobic respiration and H_2_O_2_ resistance factors. The significance underlying the regulatory tethering of these diverse processes is not clear. However, SrrAB is responsive to cellular respiratory status [36]. Cellular respiration is directly connected to cellular energy homeostasis. Therefore, one explanation would be that *S*. *aureus* reprograms its virulence repertoire in accordance with respiratory status and the energetic demands or energetic potential of the cell. However, this idea remains to be tested.

In summary, the results presented in this study show that SrrAB manages the mutually inclusive transcription of genes involved in aerobic respiration and H_2_O_2_ resistance. Our data suggest that SrrAB alters expression of H_2_O_2_ resistance factors in response to cellular respiratory status. This regulatory tuning imparts protection to AcnA from H_2_O_2_ damage and we propose that it is likely to also facilitate the adaptation of *S*. *aureus* to shifts in dioxygen concentrations and changes in respiratory flux.

## Supporting Information

S1 Fig A *srrAB* mutant strain displays increased killing by H_2_O_2_. Panel A; The lag-time necessary for *S*. *aureus* to initiate outgrowth is increased as a function of H_2_O_2_ concentration. The WT (JMB1100) strain was cultured aerobically and then diluted into fresh defined medium and challenged with varying concentrations of H_2_O_2_. Panel B; The lag-times necessary for the Δ*srrAB* strain to initiate outgrowth post H_2_O_2_ challenge is greater than the lag-times required for the WT strain. The WT (JMB1100) and Δ*srrAB* (JMB1467) strains were diluted into fresh defined medium and challenged with various concentrations of H_2_O_2_. The difference in lag-phase (relative to the WT) was determined by measuring the time to grow to an optical density (OD) of 0.2 (A_600_) for each strain and subtracting these values from the time taken for WT to grow to the same OD. Panel C; The WT (JMB1100) with pCM28 (empty vector; pEV) and the Δ*srrAB* strain (JMB1467) with pEV or pCM28_*srrAB* (*srrAB* p*srrAB*) were cultured in TSB, standardized and challenged with H_2_O_2_ for 2 hours. The H_2_O_2_ stress was terminated with catalase addition and the surviving colony-forming units (CFU) were determined. Representative data are shown in Panels A and B and experiments were repeated on three independent occasions. Data in Panel C represent average of biological triplicates with standard deviations presented for all data, but not visible in some cases. Note that the differences in H_2_O_2_ concentrations between data in Panel A and C is due to the fact that cells were adjusted to a higher optical density for the survival assay.
(TIF)

S2 Fig SrrAB is not required for the induction of H_2_O_2_ resistance genes upon H_2_O_2_ challenge. The accumulation of mRNA transcripts corresponding to H_2_O_2_ resistance genes is similar in the WT and Δ*srrAB* strains upon hydrogen peroxide challenge. The WT (JMB1100) and Δ*srrAB* (JMB1467) strains were cultured to an optical density (OD) of 6.5 (A_600_) at a HVR of 6 and challenged with 10 mM H_2_O_2_ or vehicle control. The mRNA transcript abundances corresponding to *kat* and *ahpC* were assessed post H_2_O_2_ treatment. The data were normalized to 16s rRNA transcript and are presented as a ratio of the transcript abundance upon challenge with ROS to the transcript abundance of the non-treated control for each strain. Data represent the average of biological triplicates with standard deviations shown. Two-tail student t-tests were performed on all samples *P>* 0.05 and is denoted as non-significant (NS).
(TIF)

S3 Fig SrrAB and PerR influence Kat activity independent of each another. Kat activity was assessed in cell-free lysates from the WT (JMB1100), Δ*srrAB* (JMB1467), Δ*perR* (JMB2151), and Δ*perR* Δ*srrAB* (JMB2615) strains.
(TIF)

S4 Fig Putative binding site that is conserved in promoter regions bound by SrrA. Depiction of the conserved inverted repeat sequence (bold and underlined) found in promoter regions bound by SrrA. The inverted repeat sequence was found to be separated by a variable spacer region of between 3–6 base-pairs. The sequence within the proposed *dps* promoter region had one mismatch (blue font).
(TIF)

S5 Fig Introduction of a *cidA*::*Tn* or *cidB*::*Tn* mutation into a Δ*srrA*B strain does not provide it increased protection against H_2_O_2_ intoxication in USA300_LAC. The WT (JMB1100), Δ*srrAB* (JMB1467), Δ*srrAB cidB*::*Tn* (JMB6024), Δ*srrAB cidA*::*Tn* (JMB6070), Δ*perR* (JMB2151), and Δ*kat* (JMB2078) strains were cultured aerobically, diluted into fresh TSB medium, and challenged with H_2_O_2_ at the point of inoculation. Representative growth profiles are presented and experiments were performed on least three independent occasions.
(TIF)

S6 Fig *sufC* transcriptional activity is the same in the WT and *srrAB* strains during aerobic growth. The transcriptional activity of the *sufC* gene was assessed in the WT (JMB1100) and Δ*srrAB* (JMB1467) strains containing *gfp* under the transcriptional control of the *sufC* promoter (pCM11_*sufC*). Data represent the average of biological triplicates with standard deviations shown.
(TIF)

S1 Table Oligonucleotides used in this study (Locus numbers of genes indicated in brackets or in primer names). (DOCX)
