# Supplementary figures and images for: The Staphylococcus aureus SrrAB Regulatory System Modulates Hydrogen Peroxide Resistance Factors, Which Imparts Protection to Aconitase during Aerobic Growth
Source: PLoS One. 2017 Jan 18;12(1):e0170283. doi: 10.1371/journal.pone.0170283 (PMC5242492; doi:10.1371/journal.pone.0170283)

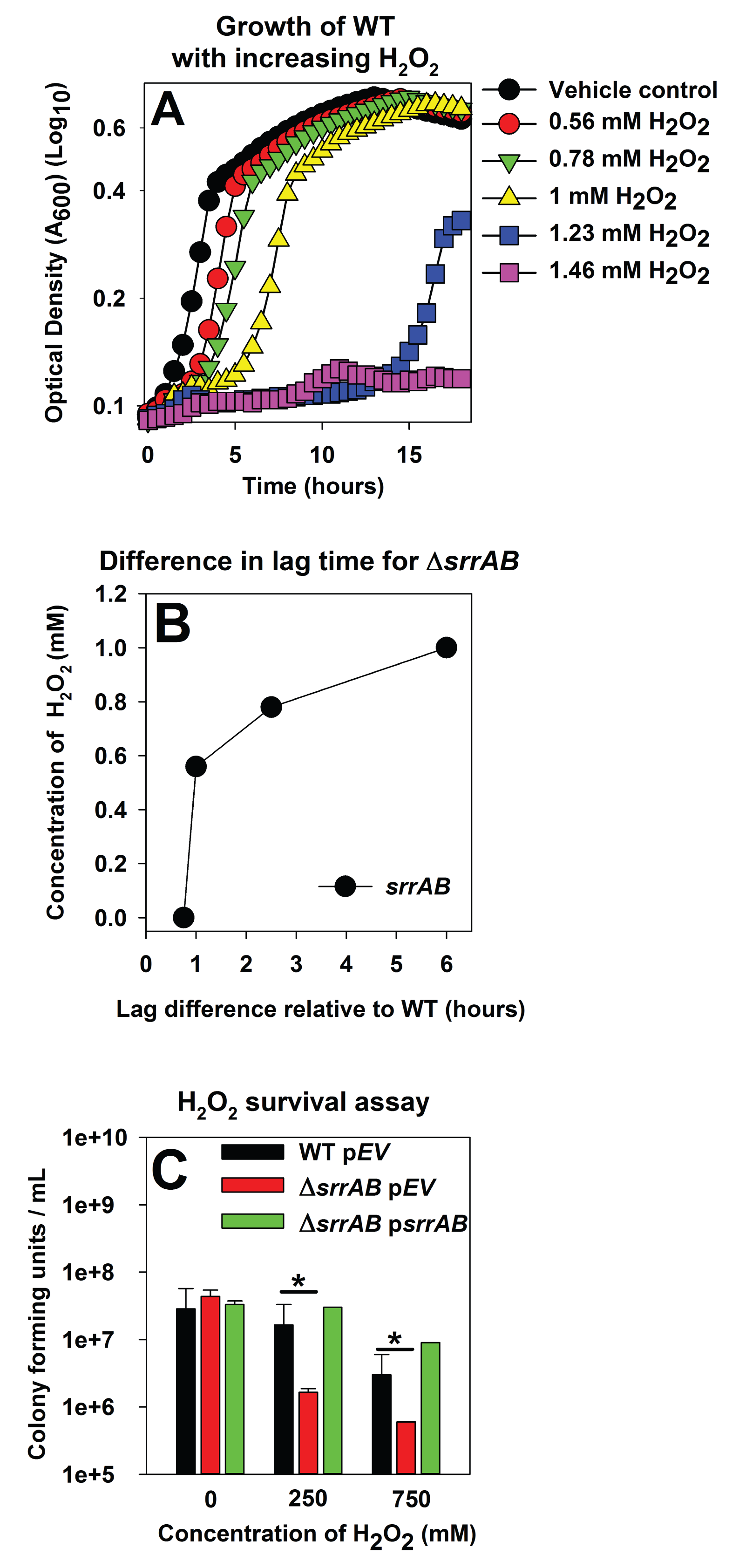

Supplement: S1 Fig — Panel A; The lag-time necessary for S. aureus to initiate outgrowth is increased as a function of H2O2 concentration. The WT (JMB1100) strain was cultured aerobically and then diluted into fresh defined medium and challenged with varying concentrations of H2O2. Panel B; The lag-times necessary for the ΔsrrAB strain to initiate outgrowth post H2O2 challenge is greater than the lag-times required for the WT strain. The WT (JMB1100) and ΔsrrAB (JMB1467) strains were diluted into fresh defined medium and challenged with various concentrations of H2O2. The difference in lag-phase (relative to the WT) was determined by measuring the time to grow to an optical density (OD) of 0.2 (A600) for each strain and subtracting these values from the time taken for WT to grow to the same OD. Panel C; The WT (JMB1100) with pCM28 (empty vector; pEV) and the ΔsrrAB strain (JMB1467) with pEV or pCM28_srrAB (srrAB psrrAB) were cultured in TSB, standardized and challenged with H2O2 for 2 hours. The H2O2 stress was terminated with catalase addition and the surviving colony-forming units (CFU) were determined. Representative data are shown in Panels A and B and experiments were repeated on three independent occasions. Data in Panel C represent average of biological triplicates with standard deviations presented for all data, but not visible in some cases. Note that the differences in H2O2 concentrations between data in Panel A and C is due to the fact that cells were adjusted to a higher optical density for the survival assay. (TIF) [file pone.0170283.s001.tif]

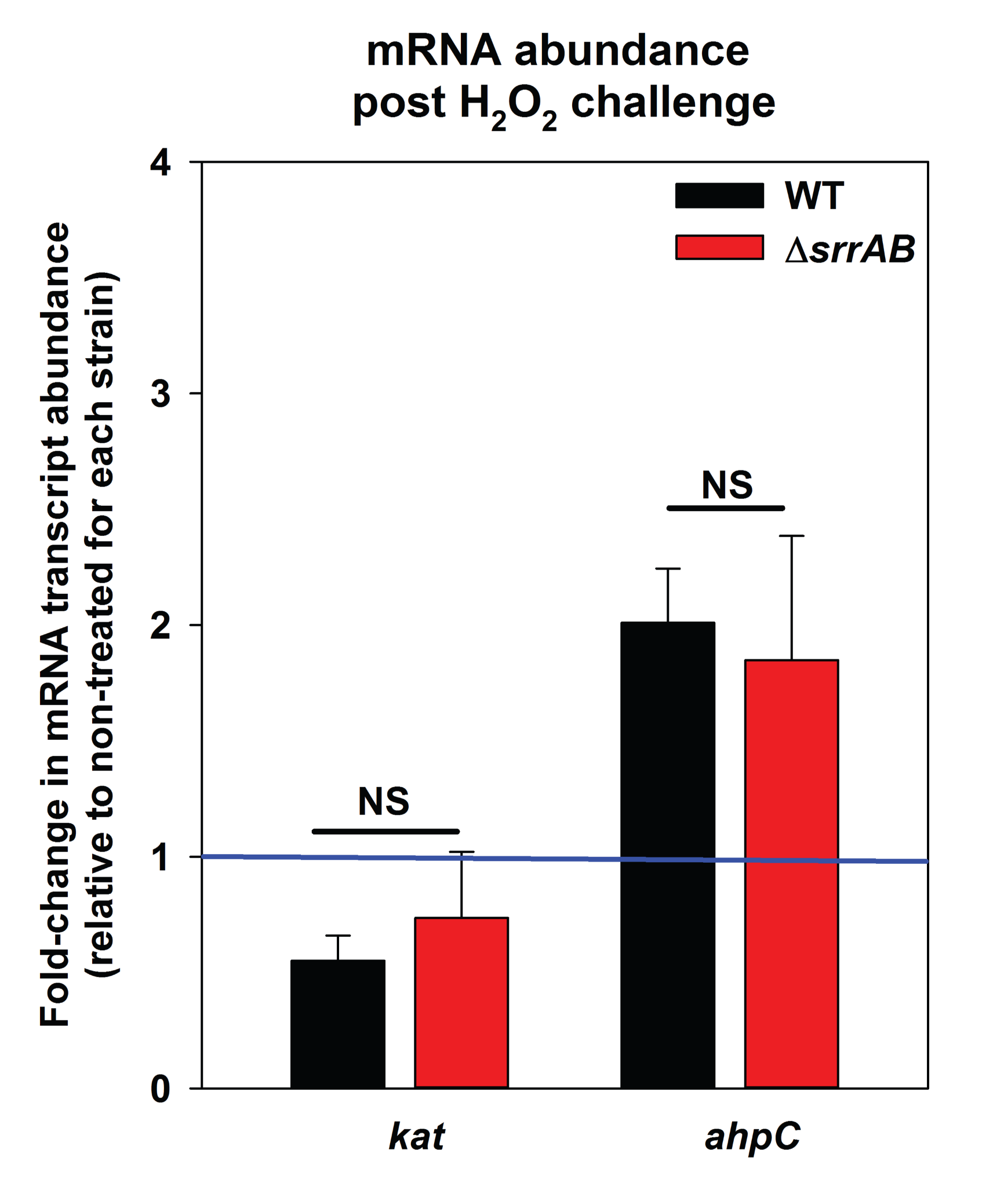

Supplement: S2 Fig — The accumulation of mRNA transcripts corresponding to H2O2 resistance genes is similar in the WT and ΔsrrAB strains upon hydrogen peroxide challenge. The WT (JMB1100) and ΔsrrAB (JMB1467) strains were cultured to an optical density (OD) of 6.5 (A600) at a HVR of 6 and challenged with 10 mM H2O2 or vehicle control. The mRNA transcript abundances corresponding to kat and ahpC were assessed post H2O2 treatment. The data were normalized to 16s rRNA transcript and are presented as a ratio of the transcript abundance upon challenge with ROS to the transcript abundance of the non-treated control for each strain. Data represent the average of biological triplicates with standard deviations shown. Two-tail student t-tests were performed on all samples P> 0.05 and is denoted as non-significant (NS). (TIF) [file pone.0170283.s002.tif]

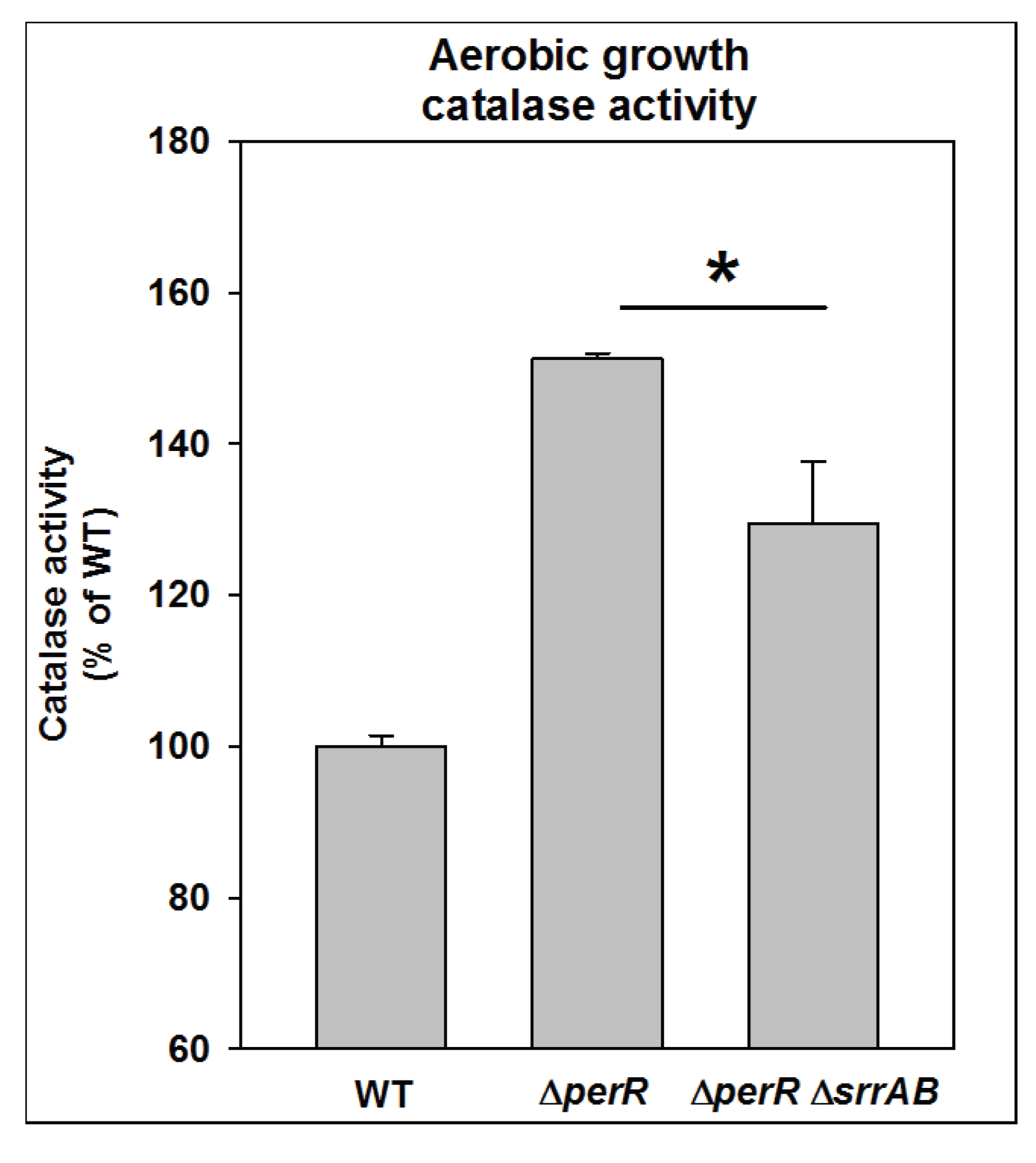

Supplement: S3 Fig — Kat activity was assessed in cell-free lysates from the WT (JMB1100), ΔsrrAB (JMB1467), ΔperR (JMB2151), and ΔperR ΔsrrAB (JMB2615) strains. (TIF) [file pone.0170283.s003.tif]

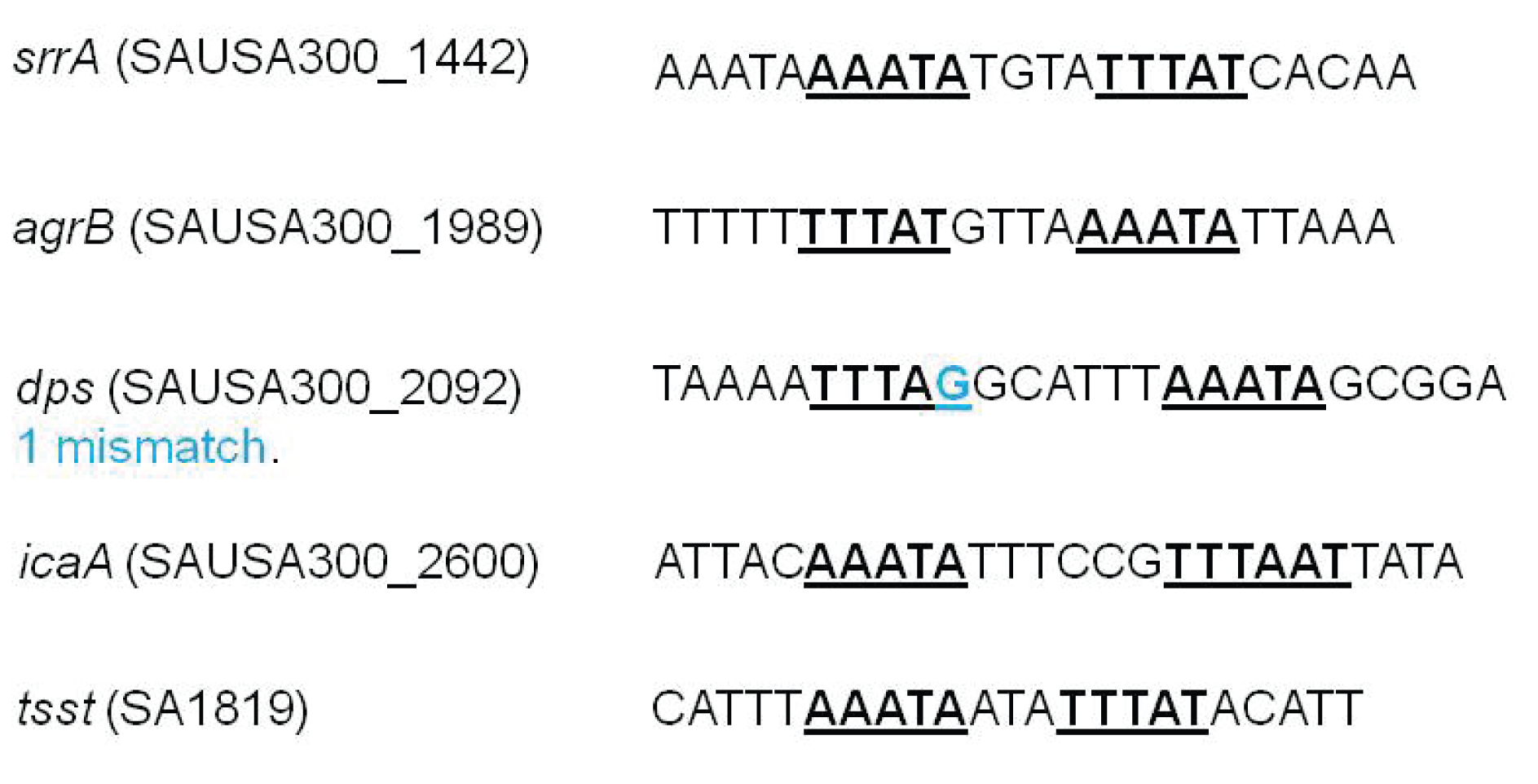

Supplement: S4 Fig — Depiction of the conserved inverted repeat sequence (bold and underlined) found in promoter regions bound by SrrA. The inverted repeat sequence was found to be separated by a variable spacer region of between 3–6 base-pairs. The sequence within the proposed dps promoter region had one mismatch (blue font). (TIF) [file pone.0170283.s004.tif]

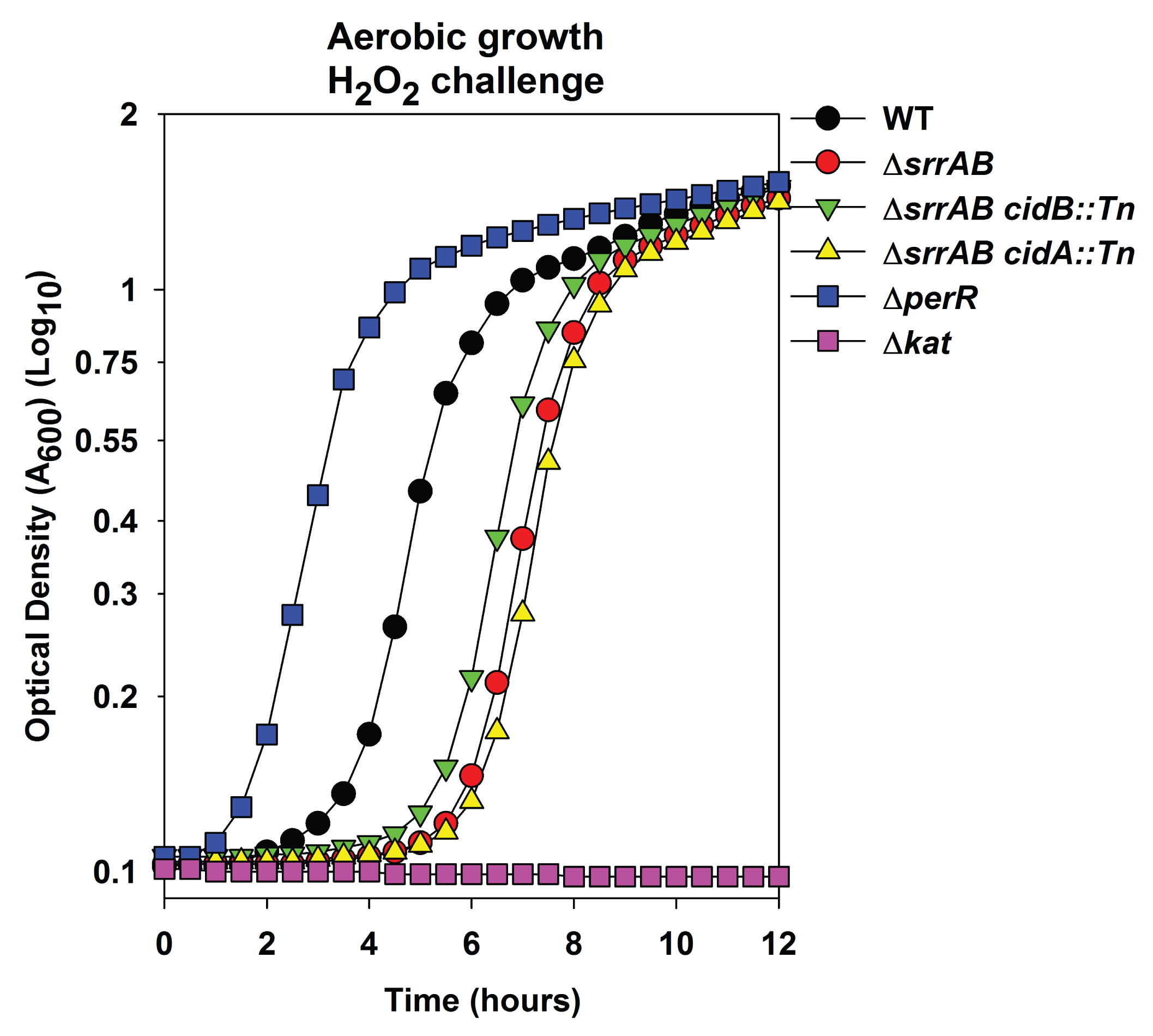

Supplement: S5 Fig — The WT (JMB1100), ΔsrrAB (JMB1467), ΔsrrAB cidB::Tn (JMB6024), ΔsrrAB cidA::Tn (JMB6070), ΔperR (JMB2151), and Δkat (JMB2078) strains were cultured aerobically, diluted into fresh TSB medium, and challenged with H2O2 at the point of inoculation. Representative growth profiles are presented and experiments were performed on least three independent occasions. (TIF) [file pone.0170283.s005.tif]

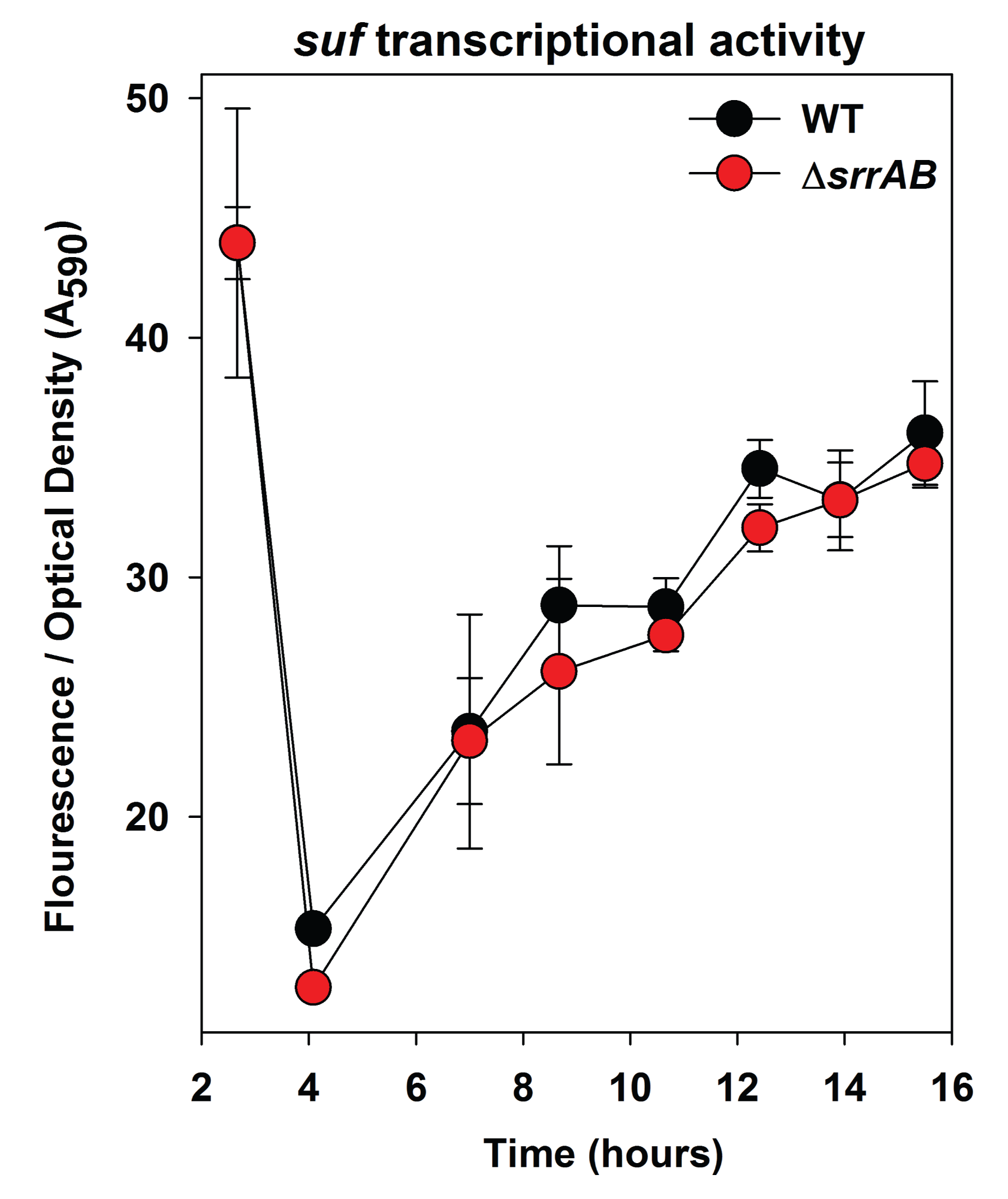

Supplement: S6 Fig — The transcriptional activity of the sufC gene was assessed in the WT (JMB1100) and ΔsrrAB (JMB1467) strains containing gfp under the transcriptional control of the sufC promoter (pCM11_sufC). Data represent the average of biological triplicates with standard deviations shown. (TIF) [file pone.0170283.s006.tif]
