# Supplementary material for: The Staphylococcus aureus SrrAB Regulatory System Modulates Hydrogen Peroxide Resistance Factors, Which Imparts Protection to Aconitase during Aerobic Growth
Source: PLoS One. 2017 Jan 18;12(1):e0170283. doi: 10.1371/journal.pone.0170283 (PMC5242492; doi:10.1371/journal.pone.0170283)
Supplement: S1 Table — (DOCX) [file pone.0170283.s007.docx]

| **RT- PCR primers** | |
| --- | --- |
| *ahp* RT For (0380) | CGTAAAAACCCTGGCGAAGTAT |
| *ahp* RT Rev | TGCAATGTTTTAGCGCCTTCT |
| *kat* RT For (1232) | TGGTGTTTTTGGGCATCCA |
| *kat* RT Rev | CCCTAGGCCCTGCTGTCATA |
| *cydB* For (0986) | GCCTTGGATTGTTCGTGGTT |
| *cydB* Rev | CCGCCTGCTTGTGTTGCT |
| *ytfe* RT For (0253) | GGCATGTTAGAGCATATGCAAAAA |
| *ytfe* RT Rev | CTACCTCACCTTGCTCATATTTAATGA |
| *dps RT For (2092)* | TCCAGAAGCGGCAAATAAAAA |
| *dps RT Rev* | GCCTGCGATTCAAAATATATACGA |
|  |  |
| **Transcriptional reporter primers** | |
| Sufgfp5hindIII | GGGAAGCTTCTCGTTCCCATAGCAAAACCTTTTAG |
| Sufgfp3kpnI | GGGggtaccCCGTAAAATATAAAGTTTTCTTAACTAG |
| 2092hindIII (2092) | CCCAAGCTTGCCAATCGTATATTATGATGGAAACTG |
| 2092kpnI | GGGGGTACCCACTCCTTAAAATTGTCTACGTCTTGC |
|  |  |
| **EMSA primers** |  |
| rpsc For (2198) | TAACGAAGGACCAACATTAAAACGTTTCCGTCCAC |
| rpsc Rev | AGT ATT CCC TCC TTA AAA GTT AAT TAA GCT TCT TTA GCT TCT |
| srrA For (1442) | GCAGAACATGGGAAATAATTAAATAAAATATGTATTTATCACAAAG |
| srrA Rev | ACA GGT CAT ACC TCC CAC ACA TGC TTT |
| *dps*  For (2092) | ATTTAGGCATTTAAATAGCGGAACACTTTACGGTTA |
| dps Rev | ATT TAA TAC ACT CCT TAA AAT TGT CTA CGT CTT GCT TG |
|  |  |
| **Cloning primers** |  |
| G+tetnheI | CCCgctagcCGGATTTTATGACCGATGATGAAG |
| G+tetmluI | CCCacgcgtTTAGAAATCCCTTTGAGAATGTTT |
| *srr*comp5BamHI | CCCGGATCCGATGGTATGACGCAACCGGCTGAAGTG |
| *srr*comp3PstI | CCCCTGCAGCCCCACATCAATCAAAAAACGCATGC |
| 0253up 5EcoRI | GGGGAATTCCTGCAGAAACAAGTGCATTAATTCATTTGAGCAG |
| 0253up3NheI | ACGCGTGGTACCGCTAGCGCTAGCCCTCCTCATTTCCAGTGTATACAATTTAA |
| 0253down5MluI | GCTAGCGGTACCACGCGTACGCGTCGCGATTAGAAACTGTTGGCAAAAATAAGTCC |
| 0253down 3KpnI | CCCGGTACCGTGTTAGCTAAAGATACATCATCATCTAAACGG |
| 2092up5EcoRI | GGGGAATTCGGCCAACAATTTTTTAAACAACATCCAGAAGCGGC |
| 2092up3NheI | ACGCGTGGTACCGCTAGCGCTAGCCACTCCTTAAAATTGTCTACGTCTTGC |
| 2092down5MluI | GCTAGCGGTACCACGCGTACGCGTGCCAACACATAAATGGGGATACGCCCAGAATG |
| 2092down3SalI | CCCGTCGACCGCGCCTATCGCTGACTTCGAGTTAGTAACTAAAGC |
| srrApET5XbaI | GGGTCTAGAAAGGAGAatgtcgaacgaaatacttatcgtagatgatgagg |
| srrApET3XhoI | GGGctcgagtttagccggctcatcattagatttaacctc |
